# Compositional response of Amazon forests to climate change

**DOI:** 10.1111/gcb.14413

**Published:** 2018-11-08

**Authors:** Adriane Esquivel‐Muelbert, Timothy R. Baker, Kyle G. Dexter, Simon L. Lewis, Roel J. W. Brienen, Ted R. Feldpausch, Jon Lloyd, Abel Monteagudo‐Mendoza, Luzmila Arroyo, Esteban Álvarez-Dávila, Niro Higuchi, Beatriz S. Marimon, Ben Hur Marimon-Junior, Marcos Silveira, Emilio Vilanova, Emanuel Gloor, Yadvinder Malhi, Jerôme Chave, Jos Barlow, Damien Bonal, Nallaret Davila Cardozo, Terry Erwin, Sophie Fauset, Bruno Hérault, Susan Laurance, Lourens Poorter, Lan Qie, Clement Stahl, Martin J. P. Sullivan, Hans ter Steege, Vincent Antoine Vos, Pieter A. Zuidema, Everton Almeida, Edmar Almeida de Oliveira, Ana Andrade, Simone Aparecida Vieira, Luiz Aragão, Alejandro Araujo‐Murakami, Eric Arets, Gerardo A. Aymard C, Christopher Baraloto, Plínio Barbosa Camargo, Jorcely G. Barroso, Frans Bongers, Rene Boot, José Luís Camargo, Wendeson Castro, Victor Chama Moscoso, James Comiskey, Fernando Cornejo Valverde, Antonio Carlos Lola da Costa, Jhon del Aguila Pasquel, Anthony Di Fiore, Luisa Fernanda Duque, Fernando Elias, Julien Engel, Gerardo Flores Llampazo, David Galbraith, Rafael Herrera Fernández, Eurídice Honorio Coronado, Wannes Hubau, Eliana Jimenez‐Rojas, Adriano José Nogueira Lima, Ricardo Keichi Umetsu, William Laurance, Gabriela Lopez‐Gonzalez, Thomas Lovejoy, Omar Aurelio Melo Cruz, Paulo S. Morandi, David Neill, Percy Núñez Vargas, Nadir C. Pallqui Camacho, Alexander Parada Gutierrez, Guido Pardo, Julie Peacock, Marielos Peña‐Claros, Maria Cristina Peñuela‐Mora, Pascal Petronelli, Georgia C. Pickavance, Nigel Pitman, Adriana Prieto, Carlos Quesada, Hirma Ramírez‐Angulo, Maxime Réjou‐Méchain, Zorayda Restrepo Correa, Anand Roopsind, Agustín Rudas, Rafael Salomão, Natalino Silva, Javier Silva Espejo, James Singh, Juliana Stropp, John Terborgh, Raquel Thomas, Marisol Toledo, Armando Torres‐Lezama, Luis Valenzuela Gamarra, Peter J. van de Meer, Geertje van der Heijden, Peter van der Hout, Rodolfo Vasquez Martinez, Cesar Vela, Ima Célia Guimarães Vieira, Oliver L. Phillips

**Affiliations:** ^1^ School of Geography University of Leeds Leeds UK; ^2^ Royal Botanic Garden of Edinburgh Edinburgh UK; ^3^ School of Geosciences University of Edinburgh Edinburgh UK; ^4^ Department of Geography University College London London UK; ^5^ Geography College of Life and Environmental Sciences University of Exeter Exeter UK; ^6^ Department of Life Sciences Imperial College London Ascot UK; ^7^ Jardín Botánico de Missouri Oxapampa Peru; ^8^ Universidad Nacional de San Antonio Abad del Cusco Cusco Peru; ^9^ Universidad Autónoma Gabriel Rene Moreno El Vallecito Santa Cruz Bolivia; ^10^ Red para la Mitigación y Adaptación al Cambio Climático - Red MiA Escuela ECAPMA de la Universidad Nacional Abierta y a Distancia Bogotá Colombia; ^11^ Instituto Nacional de Pesquisas da Amazônia ‐ Coordenação de Pesquisas em Silvicultura Tropical Manaus Brazil; ^12^ Universidade do Estado de Mato Grosso Mato Grosso Nova Xavantina Brazil; ^13^ Universidade Federal do Acre Museu Universitário Acre Brazil; ^14^ Universidad de los Andes Merida Venezuela; ^15^ School of Environmental and Forest Sciences University of Washington Seattle, Washington; ^16^ Environmental Change Institute School of Geography and the Environment University of Oxford Oxford UK; ^17^ Laboratoire Evolution et Diversité Biologique (EDB) UMR 5174 CNRS/UPS Bâtiment 4R1 Toulouse France; ^18^ Lancaster Environment Centre Lancaster University Lancaster UK; ^19^ Museu Paraense Emilio Goeldi Belém Brazil; ^20^ AgroParisTech INRA UMR Silva Université de Lorraine Nancy France; ^21^ Facultad de Ciencias Biológicas Universidad Nacional de la Amazonía Peruana Iquitos Peru; ^22^ Smithsonian Institution Washington District of Columbia; ^23^ Cirad UR Forests & Societies University of Montpellier Montpellier France; ^24^ INPHB Institut National Polytechnique Félix Houphouët‐Boigny Yamoussoukro Ivory Coast; ^25^ Centre for Tropical Environmental and Sustainability Science (TESS) and College of Marine and Environmental Sciences James Cook University Cairns Queensland Australia; ^26^ Forest Ecology and Forest Managment group Wageningen University and Research Wageningen The Netherlands; ^27^ INRA UMR EcoFoG AgroParisTech CNRS Cirad Université des Antilles Université de Guyane Kourou France; ^28^ Naturalis Biodiversity Center Leiden The Netherlands; ^29^ Systems Ecology Free University Amsterdam Netherlands; ^30^ Centro de Investigación y Promoción del Campesinado ‐ Norte Amazónico Riberalta Bolivia; ^31^ Universidad Autónoma del Beni Riberalta Bolivia; ^32^ Programa Manejo de Bosques de la Amazonía Boliviana Riberalta Bolivia; ^33^ Instituto de Biodiversidade e Floresta Universidade Federal do Oeste do Pará Pará Brazil; ^34^ Instituto Nacional de Pesquisas da Amazônia Projeto Dinâmica Biológica de Fragmentos Florestais Manaus Brazil; ^35^ Universidade Estadual de Campinas Campinas, São Paulo Brazil; ^36^ National Institute for Space Research (INPE) São José dos Campos, São Paulo Brazil; ^37^ Museo de Historia Natural Noel Kempff Mercado Universidad Autónoma Gabriel René Moreno Santa Cruz Bolivia; ^38^ Wageningen Environmental Research Wageningen University and Research Wageningen The Netherlands; ^39^ UNELLEZ‐Guanare Programa de Ciencias del Agro y el Mar Herbario Universitario (PORT) Barinas Venezuela; ^40^ International Center for Tropical Botany Department of Biological Sciences Florida International University Miami Florida; ^41^ Universidade de São Paulo São Paulo Brazil; ^42^ Universidade Federal do Acre Rio Branco, Acre Brazil; ^43^ 31 Tropenbos International and Group Ecology and Biodiversity Beta Faculty Utrecht University Utrecht The Netherlands; ^44^ Programa de Pós‐Graduação Ecologia e Manejo de Recursos Naturais Universidade Federal do Acre Rio Branco, Acre Brazil; ^45^ Jardin Botanico de Missouri Cusco Peru; ^46^ Inventory and Monitoring Program National Park Service Fredericksburg Virginia; ^47^ Andes to Amazon Biodiversity Program Puerto Maldonado Peru; ^48^ Universidade Federal do Pará Pará Brazil; ^49^ Instituto de Investigaciones de la Amazonia Peruana Iquitos Peru; ^50^ Michigan Tech University Houghton Michigan; ^51^ University of Texas at Austin Austin Texas; ^52^ AMAP IRD CIRAD CNRS INRA Boulevard de la Lironde Université de Montpellier Montpellier France; ^53^ Universidad Nacional Jorge Basadre de Grohmann (UNJBG) Tacna Peru; ^54^ Centro de Ecologia Instituto Venezolano de Investigaciones Cientificas Caracas Venezuela; ^55^ ReforeST Group DIHMA Universidad Politécnica de Valencia Valencia Spain; ^56^ Royal Museum for Central Africa Tervuren Belgium; ^57^ Grupo de Investigación en Temas Agroambientales INTEGRA Tecnológico de Antioquia Institución Universitaria Medellin Colombia; ^58^ Instituto Nacional de Pesquisas da Amazônia Manaus Brazil; ^59^ Centre for Tropical Environmental and Sustainability Science (TESS) and College of Science and Engineering James Cook University Cairns Queensland Australia; ^60^ Center for Biodiversity and Sustainability at George Mason University Fairfax Virginia; ^61^ Universidad de Tolima Villeta Colombia; ^62^ Universidad Estatal Amazónica Puyo, Pastaza Ecuador; ^63^ Universidad Regional Amazónica ikiam Tena Ecuador; ^64^ Cirad UMR Ecofog (AgrosParisTech, CNRS, INRA, Univ Guyane) Kourou French Guiana; ^65^ Science and Education The Field Museum Chicago Illinois; ^66^ Instituto de Ciencias Naturales Universidad Nacional de Colombia Bogota Colombia; ^67^ UMR 5174 Evolution et Diversité Biologique Université Paul Sabatier CNRS Toulouse France; ^68^ Servicios Ecosistémicos y Cambio Climático (SECC) Fundación Con Vida & Corporación COL‐TREE Medellin Colombia; ^69^ Iwokrama International Centre for Rainforest Conservation and Development Georgetown Guyana; ^70^ Pós‐Graduação em Botânica Tropical Universidade Federal Rural da Amazônia/Museu Paraense Emílio Goeldi Pará Brazil; ^71^ Programa de Pós‐Graduação em Agricultura e Ambiente Universidade Estadual do Maranhão São Luís, Maranhão Brasil; ^72^ Serviço Florestal Brasileiro Santarem Brazil; ^73^ Departamento de Biología Universidad de La Serena La Serena Chile; ^74^ Guyana Forestry Commission Georgetown Guyana; ^75^ Institute of Biological and Health Sciences Federal University of Alagoas Maceió Alagoas Brazil; ^76^ Center for Tropical Conservation Nicholas School of the Environment Duke University Durham North Carolina; ^77^ INDEFOR Universidad de Los Andes Mérida Venezuela; ^78^ Forest and Nature Management Group Van Hall Larenstein University of Applied Sciences Velp The Netherland; ^79^ University of Nottingham Nottingham UK; ^80^ van der Hout Forestry Consulting Rotterdam The Netherlands; ^81^ Escuela Profesional de Ingeniería Forestal Universidad Nacional de San Antonio Abad del Cusco Puerto Maldonado Perú; ^82^ Museu Paraense Emílio Goeldi Pará Brazil

**Keywords:** bioclimatic niches, climate change, compositional shifts, functional traits, temporal trends, tropical forests

## Abstract

Most of the planet's diversity is concentrated in the tropics, which includes many regions undergoing rapid climate change. Yet, while climate‐induced biodiversity changes are widely documented elsewhere, few studies have addressed this issue for lowland tropical ecosystems. Here we investigate whether the floristic and functional composition of intact lowland Amazonian forests have been changing by evaluating records from 106 long‐term inventory plots spanning 30 years. We analyse three traits that have been hypothesized to respond to different environmental drivers (increase in moisture stress and atmospheric CO_2_ concentrations): maximum tree size, biogeographic water‐deficit affiliation and wood density. Tree communities have become increasingly dominated by large‐statured taxa, but to date there has been no detectable change in mean wood density or water deficit affiliation at the community level, despite most forest plots having experienced an intensification of the dry season. However, among newly recruited trees, dry‐affiliated genera have become more abundant, while the mortality of wet‐affiliated genera has increased in those plots where the dry season has intensified most. Thus, a slow shift to a more dry‐affiliated Amazonia is underway, with changes in compositional dynamics (recruits and mortality) consistent with climate‐change drivers, but yet to significantly impact whole‐community composition. The Amazon observational record suggests that the increase in atmospheric CO_2_ is driving a shift within tree communities to large‐statured species and that climate changes to date will impact forest composition, but long generation times of tropical trees mean that biodiversity change is lagging behind climate change.

## 1 INTRODUCTION

Tropical forests represent the world's most biodiverse ecosystems, as well as providing its largest stores of living carbon and contributing more to biomass productivity than any other biome on the planet. Changes here can therefore have global consequences, potentially nowhere more so than in Amazonia where between 6,000 and 16,000 tree species exist (Cardoso et al., 2017; Ter Steege et al., 2013) and as much as 100 Pg of carbon is stored in biomass (Feldpausch et al., 2012). While the physical, chemical, and biological environment have all been changing over recent decades, it is the changes in climate—both documented and projected—which are widely expected to cause some of the most profound changes in forest communities and ecosystem processes (Esquivel‐Muelbert, Baker, et al., 2017; Thomas et al., 2004). For example, higher temperatures and intensifying drought may threaten larger trees due to hydraulic failure (McDowell & Allen, 2015; Rowland et al., 2015), which could eventually compromise forest biomass and productivity. In Amazonia, because forest diversity is concentrated in the wetter, least seasonal forests (Francis & Currie, 2003; Gentry, 1988), a persistent lengthening of the dry season might threaten a large portion of tropical biodiversity. Here we aim to better understand how Amazonian trees have responded to the last 30 years of environmental change, by analysing floristic records from long‐term tree monitoring in the Neotropics to assess the potential compositional changes to date.

Changes in biodiversity attributed to climate change have already been documented in a wide variety of ecosystems (e.g. Bowler et al., 2017; Chen et al., 2009), including in some tropical locations, but so far there is remarkably little evidence of widespread impacts of climate change on the composition of tropical ecosystems which harbour much of the planet's diversity (Duque, Stevenson, & Feeley, 2015; Fauset et al., 2012; Van Der Sande et al., 2016). In contrast, there is evidence for widespread changes in the structure (i.e. aboveground biomass) and dynamics (e.g. mortality and productivity) of old‐growth tropical forests. In many forests, apparently undisturbed by humans, both aboveground biomass and the rate of ecological processes such as growth and recruitment have increased (e.g. Brienen et al., 2015; Lewis, Lopez‐Gonzalez, et al., 2009; Phillips & Gentry, 1994; Qie et al., 2017), while in Amazonia increases in mortality have caused a recent weakening of the biomass carbon sink (Brienen et al., 2015). However, it remains unclear whether these structural and dynamic changes are also associated with concerted changes in the species richness and composition of Amazonian forests.

In Amazonia, as elsewhere, climate change is a potential leading driver of changes to the ecosystem. During the last few decades, the climate of Amazonia has become more extreme—the length of the dry season and its intensity have increased, while precipitation has become more intense during the wet season (Gloor et al., 2015; Hilker et al., 2014). Extreme climate events in recent years include the three strong droughts within a decade (Erfanian, Wang, & Fomenko, 2017; Jiménez‐Muñoz et al., 2016; Lewis, Brando, Phillips, Van Der Heijden, & Nepstad, 2011; Marengo, Tomasella, Alves, Soares, & Rodriguez, 2011; Marengo et al., 2008) and several large‐scale episodes of extreme rainfall (Espinoza et al., 2014; Marengo & Espinoza, 2016). In addition to the repeated drought events, precipitation has declined in the south and south east of the basin (25% reduction in rainfall between 2000 and 2012) (Hilker et al., 2014) and higher temperatures are likely to have intensified seasonal evaporative stress across the basin (Jiménez‐Muñoz, Sobrino, Mattar, & Malhi, 2013). These changes are consistent with model‐based predictions (Duffy, Brando, Asner, & Field, 2015), implying that the Amazon may already have entered a new regime of a hotter, more variable climate. The forest has clearly responded to these recent fluctuations in climate—for example, tree mortality rates increased markedly during and after drought events causing at least temporary losses of standing biomass (Brienen et al., 2015; Feldpausch et al., 2016; Phillips et al., 2009; Zuleta, Duque, Cardenas, Muller‐Landau, & Davies, 2017). The 2010 drought also impacted on the basin‐wide exchange of carbon dioxide between the vegetation and the atmosphere, with the vegetation becoming a net source of CO_2_ during 2010 (Gatti et al., 2014).

In addition to the changes in climate, atmospheric CO_2_ concentrations have increased globally from 320 ppm to over 400 ppm over the past 50 years (Conway & Tans, 2016). Carbon dioxide is a fundamental resource for photosynthesis, and higher concentrations are expected to stimulate plant growth (Lloyd & Farquhar, 1996). Indeed, the increase in atmospheric concentrations of CO_2_ is a potential driver of the observed increase in aboveground biomass and turnover rates in tropical forests (Brienen et al., 2015; Lewis, Lloyd, Sitch, Mitchard, & Laurance, 2009; Pan et al., 2011; Zhu et al., 2016). Additionally, under higher CO_2_ concentrations plants may increase their water‐use efficiency with less water being required per unit of carbon fixed. Thus, by allowing plants to use less water, higher CO_2_ concentrations could alleviate the effect of increasing aridity (Lloyd & Farquhar, 2008; Van Der Sleen et al., 2015).

Interpreting potential shifts in tropical floristic composition and linking them with possible drivers is a considerable challenge due to the very high diversity of tropical forests and their large spatial extent. However, if sufficient high‐quality, long‐term floristic monitoring is available, then the approach of analysing shifts in a suite of functional traits to describe how communities change over time can be used to link floristic changes to their drivers (McGill, Enquist, Weiher, & Westoby, 2006; Violle, Reich, Pacala, Enquist, & Kattge, 2014). For tropical tree species, two largely independent trait axes may have value in addressing these questions. One axis, the life‐history trade‐off between growth and mortality, can be represented by wood density as it is negatively correlated to growth and mortality rates. This is a reflection of slow‐growing trees tending to invest more in wood structure (Chave et al., 2009; Muller‐Landau, 2004; Nascimento et al., 2005; Poorter et al., 2010). The other axis is related to the potential size that taxa can attain, representing the capacity of taxa to compete for light (Falster & Westoby, 2005; Poorter, Bongers, Sterck, & Wöll, 2005).

Environmental changes could have different impacts along each of these ecological axes (Chave et al., 2008; Lewis, Lloyd, et al., 2009). Most notably, with more extended or intense periods of soil water deficit, large trees and those with low wood density may be at greatest risk of hydraulic failure due to cavitation (McDowell & Allen, 2015; Rowland et al., 2015). Large trees have been shown to be particularly affected by artificially‐imposed drought (McDowell & Allen, 2015; Nepstad, Tohver, Ray, Moutinho, & Cardinot, 2007; Rowland et al., 2015) and drought events (Bennett, Mcdowell, Allen, & Anderson‐Teixeira, 2015; Phillips et al., 2010). On the other hand, several observations from tropical forests show a decline of small understory taxa associated with increases in soil water deficit (e.g. Condit, Hubbell, & Foster, 1996; Enquist & Enquist, 2011; Fauset et al., 2012; Feeley, Davies, Perez, Hubbell, & Foster, 2011). To explain these observations, it has been hypothesized that small taxa have shallower roots and are therefore more vulnerable compared to large, deep rooted trees to long‐term drying trends (Condit et al., 1996; Fauset et al., 2012). Although the link between rooting depth and tree size is still unclear (Stahl et al., 2013), this hypothesis is consistent with wetter areas tending to have more densely populated understoreys (Malhi et al., 2002; Pitman et al., 2002) and taller forests being less sensitive to precipitation variability (Giardina et al., 2018). The ongoing increase in atmospheric carbon dioxide is also expected to cause changes in species composition as it is predicted to favour those trees that have greater competitive capacity to access light (Coomes, Lines, & Allen, 2011; Laurance, 2004), consequently increasing the mean potential tree size within the community (e.g. Laurance et al., 2004) and to favour fast‐growing trees, potentially leading to communities with lower wood density.

Given the uncertainty about how tree size relates to responses to a drying climate, the geographic distributions of species over precipitation gradients offer an alternative source of information with which to predict and to infer the effects of drought on floristic composition. The spatial distribution of tree taxa over precipitation gradients has been shown to provide a valuable proxy for drought tolerance in observational studies and experiments (Engelbrecht et al., 2007; Esquivel‐Muelbert, Galbraith, et al., 2017). If drought is increasingly affecting Amazonian forests, we might therefore expect concerted shifts in tree communities towards more dry‐affiliated components. A compositional shift towards more dry‐tolerant taxa as a consequence of an increase in moisture stress has been reported locally for sites in southern Ghana (Fauset et al., 2012), Central America (Enquist & Enquist, 2011; Feeley, Davies, et al., 2011), and parts of the Amazon (Butt et al., 2014).

**Figure 1:**
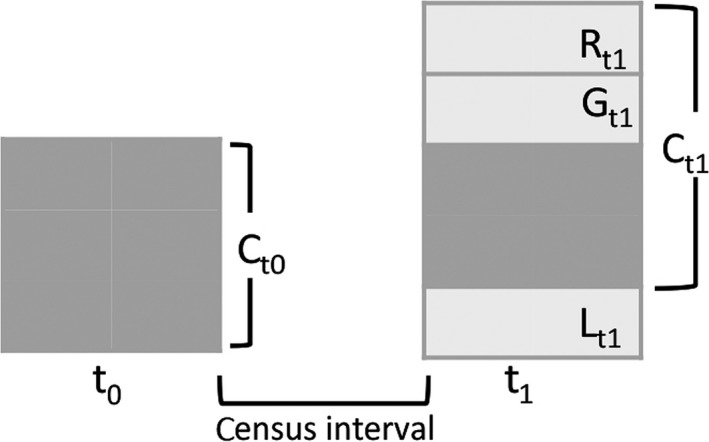
Schematic model representing the different components of forest demography. The box on the left represents an inventory plot of a forest community at the first census (*C*_t0_), while the box on the right shows the community at the second census (*C*_t1_). At *C*_t1_ recruits (*R*), that is those trees that attained 10 cm of diameter within the census interval, will now be part of the community analysed. Other trees will have died thus leaving the community, here called *losses* (*L*). Those trees from *C*_t0_ that survive through the census interval are expected to grow (*G*). Thus, the basal area of the whole community at *t*_1_ is *C*_t1_ = *C*_t0_ + *G*_t1_ + *R*_t1_ − *L*_t1_ and the net flux between *t*_0_ and *t*_1_ = *G*_t1_ + *R*_t1_ − *L*_t1_. Here we investigate the trends in the characteristics and identity of genera within these components of forest demography. This figure represents dynamics in basal area terms; similar logic can be applied for stem‐based analyses. Note that in this case we would not be interested in the growth of trees surviving from *t*_0_ to *t*_1_, and so the net flux would be represented as *R*_t1_ − *L*_t1_

Here, we aim to quantify the shifts in the floristic composition of Amazonian tree communities, and test the hypothesis that recent climatic drying is already impacting the composition of Amazonian forests. The data set derives from 106 long‐term, tree inventory plots distributed across intact closed‐canopy moist forests in lowland Bolivia, Brazil, Colombia, Ecuador, French Guiana, Guyana, Peru and Venezuela (Supporting Information Appendix S1). We analyse monitoring records from as early as 1985 onwards to as recently as 2015, deliberately excluding any possible influence of the 2015–2016 El Niño drought from our analysis. We investigate trends within the overall community composition, as well as among recruits, trees that died, and in the growth rate that occurred within each census interval (Figure 1). We analyse compositional shifts along these three trait axes, which we demonstrate to be independent: life‐history (using wood density as a proxy), potential size, and bioclimatic distribution (Supporting Information  S2). Based on predictions from plant physiology supported by experimental studies, we expect increases in dry season duration or intensity to shift floristic composition towards dry‐affiliated and smaller‐statured genera with high wood density (McDowell & Allen, 2015; Rowland et al., 2015). Additionally, we examine trends in abundance for individual genera, which allows us to understand which taxa dominate the changes in functional composition.

## 2 MATERIALS AND METHODS

### 2.1 Field observations and forest dynamics

We investigate the trends in functional and floristic composition of tree communities by analysing long‐term data from permanent tree inventory plots in the Amazon and adjacent lowland forests (Supporting Information Appendix S1). A total of 106 South American forest plots from the RAINFOR network (Malhi et al., 2002), accessed via the ForestPlots.net repository (Lopez‐Gonzalez, Lewis, Burkitt, & Phillips, 2011), met our criteria: (a) sampling lowland (<1,000 m.a.s.l), *terra firme,* intact, moist forests (i.e. where mean maximum cumulative water deficit (MCWD) over the last century is less negative than −300 mm/year); (b) having been monitored throughout the period of the two intense dry seasons in 2005 and 2010 (see Supplementary material for analyses including plots that were not monitored during 2005 and 2010); (c) having had regular monitoring, thus excluding any plots where census interval lengths differed by more than 10 years; (d) having at least 80% of tree stems identified at least to genus level. The selected plots have a mean area of 1.25 ha (95% CI = 1.16, 1.35), and have been re‐censused on average seven times, with a mean census interval of 2.8 years.

The long‐term plots were monitored following a standardized protocol (Phillips et al., 2016). Full methodological details are given elsewhere (Brienen et al., 2015). In brief, all trees and palms ≥10 cm diameter (D) at 1.3 m (or above‐buttress) are tagged, identified to the species level (when possible), have their D measured, and the point of measurement marked and recorded. At every census, trees previously recorded are re‐measured, and new recruits—that is trees that have newly attained 10 cm when the plot is revisited—tagged, measured and identified, and notes are taken about the individuals that died between censuses. Lianas and nonwoody freestanding plants (*Phenakospermum*) are excluded from our analyses.

### 2.2 Climate data

To characterize the change in moisture stress, we calculated temporal trends in maximum cumulative water deficit (MCWD—Aragão et al., 2007) for each plot. MCWD represents the most negative value of water deficit (wd), that is, the difference between precipitation (*P*) and evapotranspiration (*E*) within each year, where for each month (*n*) wd is quantified as:

(1) $$\begin{array}{cc} & \mathrm{If}\;{\mathrm{wd}}_{n-1}-E_{n}+P_{n}<0;\\ & \mathrm{then}\;{\mathrm{wd}}_{n}={\mathrm{wd}}_{n-1}-E_{n}+P_{n}\\ & \mathrm{else}\;{\mathrm{wd}}_{n}=0 \end{array}.$$

In other words, MCWD is an annual water deficit metric which takes into account both the length and intensity of the dry season based solely on climatic variables, that is, ignoring soil properties. The calculation of MCWD does not necessarily follow the calendar year, as for tropical forests in the northern hemisphere the annual dry season typically spans two calendar years (between October and March). Thus, the starting point, that is, when *n* = 1, was defined climatologically as the wettest month in the first year in the time series (i.e. 1985), rather than the first month of that calendar year.

In addition to estimating annual MCWD, following Feldpausch et al. (2016), we also estimated the intensity of the most extreme dry season between two consecutive censuses, hereafter termed MCWD_i_. This metric represents a measure of maximum environmental disruption between two censuses, that is the most negative value of annual MCWD between each successive pair of censuses. Only complete years were considered for this calculation. For the first census of each plot, the MCWD_i_ was calculated as the most negative MCWD values within the 3‐year period preceding that census. This time window is equivalent to the mean census interval within the data (2.8 years).

Climate data were obtained from the Climatic Research Unit (CRU), at 0.5^°^ spatial resolution from 1985 to 2015 (Harris, Jones, Osborn, & Lister, 2014) where evapotranspiration is calculated based on the Penman–Monteith equation (Allen, Smith, Pereira, & Perrier, 1994) using information on temperature, cloud cover and vapour pressure (Harris et al., 2014). MCWD trends calculated from this ground‐based precipitation data source were consistent with satellite‐based data from the Tropical Rainfall Measuring Mission (TRMM – Huffman et al., 2007) at 0.25^°^ spatial resolution between 1998 and 2010 (*R* ^2^ = 0.87; *p* < 0.0001—see Supporting Information Appendix S3 for more detail). Here we opt to use ground‐based data from CRU as this covers the whole time window of the floristic analyses.

### 2.3 Traits

We describe Amazonian tree genera in terms of the three basic traits shown in previous work and in Supporting Information Appendix S2 for our data to represent largely independent axes of fundamental plant characteristics and each potentially responsive to environmental change: Potential Size (PS; cm): the 95th percentile of the distribution of trees’ diameter derived from a set of 530 inventory plots across the Amazon Basin (Fauset et al., 2015); Water deficit affiliation (WDA; mm): derived from relative abundances across 513 inventory plots distributed along a large gradient of MCWD across the Western Neotropics (Esquivel‐Muelbert, Baker, et al., 2017). This metric quantifies the preferred dry season severity for each taxon and is calculated as the dry season severity (measured as MCWD) where a taxon occurs weighted by the taxon's relative abundance in each location (Esquivel‐Muelbert, Baker, et al., 2017). Taxa affiliated to dry conditions show the most negative values of WDA, while the most wet‐affiliated ones have values of WDA equal to zero; Wood density (WD; g/cm^3^): values were extracted from the Wood Density Database (Chave et al., 2009; Zanne et al., 2009). There was no indication of any pairwise relationship among any of the traits: WDA versus PS (*R* ^2^ = 8 × 10^−3^) WDA versus WD (*R* ^2^ = 5 × 10^−4^) and WD versus PS (*R* ^2^ = 2 × 10^−3^).

The diversity of Amazonian flora hinders us from performing consistent species‐level analyses as comprehensive trait data are still missing for the large majority of Amazonian tree species. Therefore, our analyses were performed at the genus level, and the mean trait value of the genus was assigned to each individual. Our approach is expected to be adequate to capture the actual functional shifts in Amazonia as it captures most of the trait variation of Amazonian trees, which is mostly accounted for at the family and genus level (see Baker et al., 2004; Coelho de Souza et al., 2016; Fyllas et al., 2009; Patiño et al., 2009). Although genus‐level analyses still miss some information on trait variation, analyses at this taxonomic resolution are also potentially more conservative with respect to the hypothesis of environmental‐driven floristic change than analyses at the species level, as they use mean genus‐level values instead of (more extreme) species‐level values. Results of analyses using species‐level traits, for those taxa where data are available, do not differ from genus‐level results (see Supporting Information Appendix S8).

Genus‐level trait data were missing for 6%, 9% and 0.04% of all stems for PS, WDA and WD, respectively. For these cases, the mean trait values from the family were used, following established conventions (Baker et al., 2004; Flores & Coomes, 2011) and considering the phylogenetic conservatism of PS and WD for Amazonian trees (Coelho de Souza et al., 2016). For the small proportion of individuals belonging to families for which no trait information was available, we used average trait values of the community in the same census was used. For those stems not identified to family level (3.9%), we applied the mean trait for all dicot individuals of the plot census during which the tree was recorded. Considering the low proportion of missing data the imputation technique is unlikely to strongly affect our results (Penone et al., 2014).

Then, to obtain a census‐level value for each of the three traits, we scaled the genus‐level traits to the community level by calculating the community‐weighted mean (CWM sensu Diaz et al., 2007) for each trait in each census. For each of the 743 censuses across 106 plots, the CWM of each of these components was calculated as the mean trait value across the genera of the community, weighted by (a) the number of stems; and (b) the total basal area occupied by each genus. Community‐weighted means were calculated for the whole community and for each component of forest dynamics, that is the recruits (new trees that reach the 10 cm D cut‐off after each census interval), losses (those trees that died within each census interval) and the basal area gain of those trees that survived the census interval (Figure 1).

### 2.4 Analytical approach

We investigated changes in functional (mean potential size, water deficit affiliation and wood density) and floristic composition (relative abundance of individuals within different genera) by assessing trends over time of these quantities for each plot and their Amazon‐level mean.

#### 2.4.1 Trends in functional composition

We investigated functional trends for the different components of forest demography (i.e. whole tree community, recruits and trees that died—Figure 1). These functional trends were quantified using (a) the bootstrapped mean and 95% CI of plot level linear regression slopes of the community weighted mean (CWM) as a function of time, averaged across all plots; and (b) linear mixed effect models (LMM) of CWM as a function of time with plot identified as a random effect, using function *lmer* from the r package *lme4* (Bates, Mächler, Bolker, & Walker, 2015). While the first approach provides information of the overall mean trend across all plots, in which the uncertainty estimate is derived using a nonparametric approach, the second approach gives an estimate of the trend over the whole time series (1985–2015), accounting for potential changes in which plots are analysed over different time windows along the 30‐year period.

The bootstrapped mean and confidence intervals were calculated from the linear slopes of CWM (i.e. mean plot level traits) for each plot where the CWM in each census (*j*) is a function of the date when the census took place (time):

(2) $${\text{CWM}}_{j}\;∼\;\beta_{1}+\beta_{2}\times {\text{time}}_{j}+\varepsilon_{j}.$$

Then, an Amazon‐wide weighted mean and the 95% CI were estimated by randomly resampling values of the plot‐level slopes (*β*_2_ from Equation (3)) across all plots, 10,000 times. We further estimate the potential influence of spatial autocorrelation on our results by testing the correlation between the Euclidean distance in the trends in CWM and the spatial distance between the plots (Supporting Information Appendix S7).

The variation in plot area and monitoring period may be expected to influence plot‐level trends as smaller plots and those monitored for shorter periods are more likely to be affected by stochastic phenomena, such as tree falls (Fisher, Hurtt, Thomas, & Chambers, 2008). An empirical investigation of the impact of sampling effort (i.e. length of monitoring period and plot size) on our estimate showed that the variance is expected to scale as a function of the square root of plot area multiplied by the monitoring period (Supporting Information Appendix S4). Thus, to account for the noise attributed to sampling effort, we used the square root of plot area multiplied by the monitoring period as weights when calculating Amazon‐level mean and confidence intervals of the CWM slopes.

The LMM follows the same logic of the previous analyses with CWM as a linear function of time:

(3) $${\text{CWM}}_{\mathrm{ij}}\;∼\;\left(\beta_{1}+a_{i}\right)+\left(\beta_{2}+b_{i}\right)\times {\text{time}}_{\mathrm{ij}}+\varepsilon_{\mathrm{ij}}.$$

where CWM in plot *i* and census *j* is a function of the date when the census occurred (time). Here time is used as a fixed effect, and the random components of the model are the intercept (a) and slope (b) for each plot and the overall residuals (*ε*). The slope and intercept of each plot were included as random effects considering that both the initial trait value (represented by the plot intercept, *a*_i_) and the intensity of change (represented by the plot slope, *b*_i_) may differ across plots. An exploration of the influence of sampling effort on the variance of the residuals shows no weighting procedure to be required for this analytical framework (Supporting Information Appendix S4).

#### 2.4.2 The influence of climate on functional composition

To explore the potential impact of climate changes on functional change, we tested whether changes in the community are related to changes in climate. First, we calculated the linear trend in MCWD and MCWD_i_ over the inventory period for each plot. We then used Kendall's *τ* coefficient to test for the correlation between linear slopes of change in CWMs (composition) and MCWD (climate) for each plot. For the cases where the correlation was significant, we fit an OLS linear regression with the trends in CWM as a function of trends in MCWD.

#### 2.4.3 Trends in floristic composition

We investigated the influence of individual taxonomic groups on any functional shifts by analysing the relationship between shifts in abundance and trait values. Trends in relative abundance for each taxon were calculated following the same procedure applied above to analyse CWM changes, that is by calculating the bootstrapped mean of linear trends and applying LMM, but here using the relative abundance of each genus as a response variable instead of CWM. The bootstrap means were calculated from a modified version of Equation (3) applied to every plot:

(4) $${\text{RA}}_{j}\;∼\;\beta_{1}+\beta_{2}\times {\text{time}}_{j}+\varepsilon_{j}.$$

where RA is the relative abundance of a genus at plot *i* and census *j*. We then use the LMM approach by modifying Equation (4) to:

(5) $${\text{RA}}_{\mathrm{ij}}\;∼\;\left(\beta_{1}+a_{i}\right)+\left(\beta_{2}+b_{i}\right)\times {\text{time}}_{\mathrm{ij}}+\varepsilon_{\mathrm{ij}}.$$

An Amazon‐wide slope using each of the methods was calculated for each taxon. The LMM analysis was restricted to genera that occurred in three or more plots. As for the functional analyses, each plot in the bootstrap mean following Equation (5) was weighted by the square root of plot area multiplied by the monitoring period and no weighting procedure was applied for the LMM approach (Equation  6). Trends in abundance were also calculated for families and species (Supporting Information Appendix S12).

Next, we investigated which genera contribute most to the significant functional changes that were detected. When trends in functional composition were significantly different from zero (see Section 2.4.1 for details), we estimated Kendall's *τ* coefficient of correlation between Amazon‐wide slopes (calculated for the whole community, for recruits, and for losses) for each genus and their trait values (WDA, WD or PS). To ensure that abundance trends were estimated with reasonable levels of uncertainty, we restricted the investigation of the trends in abundance versus traits relationship to only include genera within the upper 20% quantile of abundance across the whole data set.

Finally, we calculated the trends in abundance for four Amazonian functional types defined by Fyllas, Quesada, and Lloyd (2012) using trait information from a subset of the Amazonian species included in this study. The four Amazonian functional groups (small pioneers, small‐statured nonpioneers, tall pioneers and tall nonpioneers) are based on variety of foliar and structural traits (Supporting Information Appendix S10) independent from the traits considered in our main analyses. This therefore provides complementary information to our main analyses.

## 3 RESULTS

### 3.1 Climate trends

**Figure 2:**
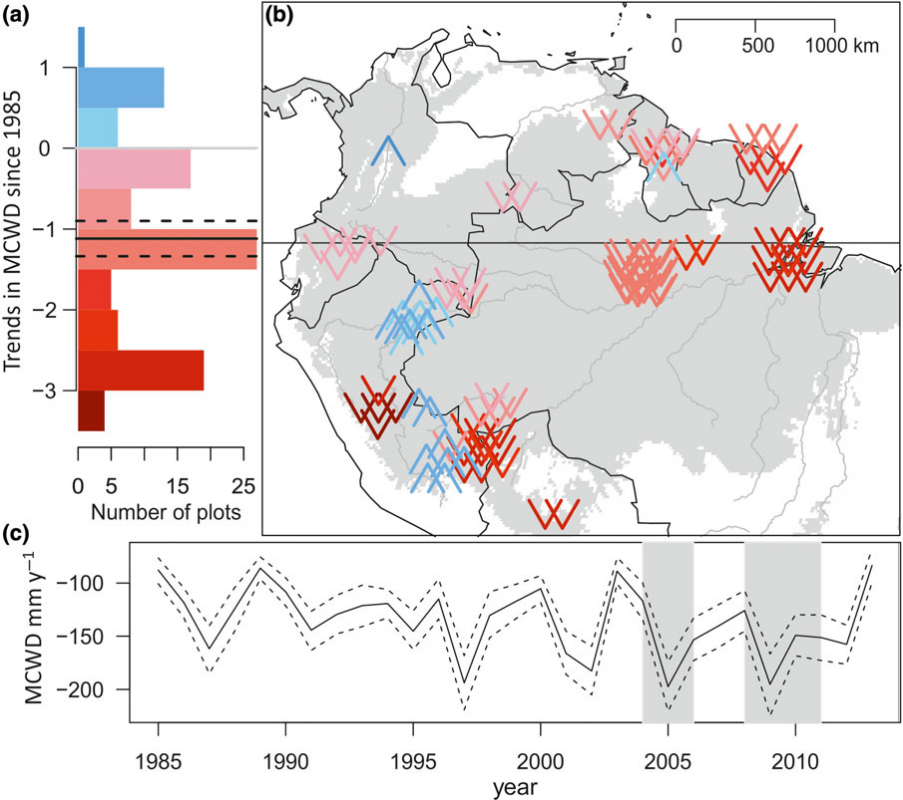
Trends in maximum cumulative water deficit (MCWD) across the Amazon Basin. (a) Frequency of annual linear trends in MCWD per plot between 1985 and 2014. Note that for most plots, the climate has significantly shifted towards more negative MCWD values. Mean change and 95% confidence intervals (black solid and dashed lines) across our plots weighting plots by plot area were calculated using a bootstrap procedure by resampling the trends in MCWD from all plots 10,000 times with replacement. (b) Distribution of annual linear trends in MCWD per plot. Arrows pointing down (in red) represent locations where MCWD has become more negative over time, that is the dry season has become more intense. Arrows pointing up (blue) represent less negative values of MCWD meaning that moisture stress decreased. The intensity of the colours in (a,b) represent the strength of the climate trend. Note the difference in scale between drying and wetting trends colour bars. (c) Mean annual MCWD across plots, and 95% CI from resampling among all plots, note lower MCWD values at 2004–2005 and 2009–2010 (grey‐shaded rectangles)

The forests analysed here have on average experienced a strengthening of the dry season, as measured by maximum cumulative water deficit (MCWD). The plot‐level annual MCWD became more negative by on average 1.1 mm/year (95% CI = 1.4, 0.9) since 1985 (Figure 2). This represents a marked intensification in MCWD across our plots by on average 36% between 1985 and 2014. There are exceptions to the overall drying trend, with some plots near the Andes becoming wetter during this period (Figure 2).

### 3.2 Trends in functional composition

Overall, there has been a significant increase in the potential size (PS) of tree communities: the community weighted mean (CWM) of PS when weighted by basal area increased by 0.03 cm/year (95% CI = 0.02, 0.05 cm/year) or 0.06% per year with basal gains increasing in PS by 0.06 cm/year (95% CI = 0.01, 0.12 cm/year) meaning that plots have been progressively occupied by larger statured genera (Table 2). The increase in basal area‐weighted community‐level PS holds regardless of the analytical technique, that is bootstrapped means or LMM (Table 2, Supporting Information Appendix S6).

**Figure 3:**
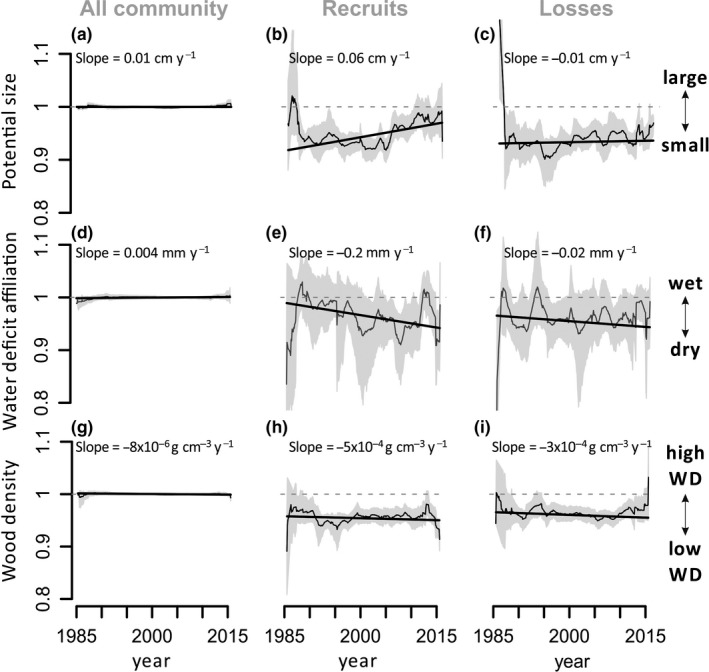
Trends in functional composition between 1985 and 2015 across Amazonia. The *y*‐axes show stem‐based community weighted mean (CWM) trends in (a–c) water deficit affiliation (WDA), (d–f) potential size (PS) and (g–i) wood density (WD) at genus level. Values are standardized with respect to the whole plot population to allow comparison among traits meaning that the value for the whole community in the first census is equal to 1. CWM trends are shown for the whole community (a,d,g), recruits (b,e,h), and losses (c,f,i). Grey line and grey‐shaded area show standardized mean and 95% CI census‐level CWM, which can be influenced by some switching of plots assessed through time. Trend lines show linear mixed models (LMM) considering the slope and intercept of plots as random effects. Slope values for LMM are shown in each graph—these are not standardized by plot population and are shown at a different scale for each trait

For the recruits, dry‐affiliated genera have become more abundant, with water‐deficit affiliation (WDA) becoming significantly more negative for this segment of the community by 0.45 mm/year (CI 95% −0.9, −0.03 mm/year) or 0.3% per year (Table 1). This trend was marginally significant across the basin when calculated using LMM (Figure 3, Supporting Information Appendix S6.1); however, it was observed for 62 of 102 plots (*p* = 0.03, two‐tailed binomial test) and persisted when our analyses were repeated across a larger data set (Supporting Information Appendix S6.2) and when grouping together nearby plots to account for spatial autocorrelation in the distribution of plots (Supporting Information Appendix S7.2).

**Table 1:** Mean linear slopes in stem‐based functional composition in Amazonia

Community	Potential size (cm/year)	Water deficit affiliation (mm/year)	Wood density (g cm^−3^ year^−1^)
All community	0.01 (−0.002|0.01)	0.01 (−0.03|0.04)	−1 × 10^−5^ (−9 × 10^−5^ |6 × 10^−5^)
Gains (recruits)	0.07 (−0.03|0.2)	−**0.45 (**−**0.9**|−**0.03)**	−3 × 10^−4^ (−2 × 10^−3^|1 × 10^−3^)
Losses	0.1 (−0.01|0.2)	−0.1 (−0.6|0.3)	2 × 10^−4^ (−7 × 10^−4^|1 × 10^−3^)
Net fluxes	−0.03 (−0.2|0.1)	−0.45 (−1|0.1)	−7 × 10^−4^ (−2 × 10^−3^|8 × 10^−4^)

*Notes*

For each trait, we show the bootstrap mean annual changes in community weighted mean (CWM) and 95% confidence intervals (CI, in brackets) weighted by the product of the squared root of plot size and monitoring period. CWM is calculated using: water deficit affiliation (WDA), potential size (PS) and wood density (WD). The analyses were repeated for recruits, losses and the difference between recruits and dead trees (net fluxes). Significant trends are in bold, that is, where 95% CIs do not overlap zero.

Among the trees that died within the study period (losses), basal area‐weighted wood density (WD) decreased by 1 × 10^−3^ g cm^−3^ year^−1^ or 0.16% per year in the LMM analyses (Supporting Information Appendix S6). This trend, however, was only observed for 53 of 102 communities, meaning that positive and negative slopes are equally likely to be observed (*p* = 0.8, two tail binomial test) and, it was not significant when using bootstrapped means (Table 1). No equivalent significant abundance‐based trend for losses in WD terms was observed (Table 1, Supporting Information Appendix S6).

### 3.3 The influence of climate on functional composition

**Figure 4:**
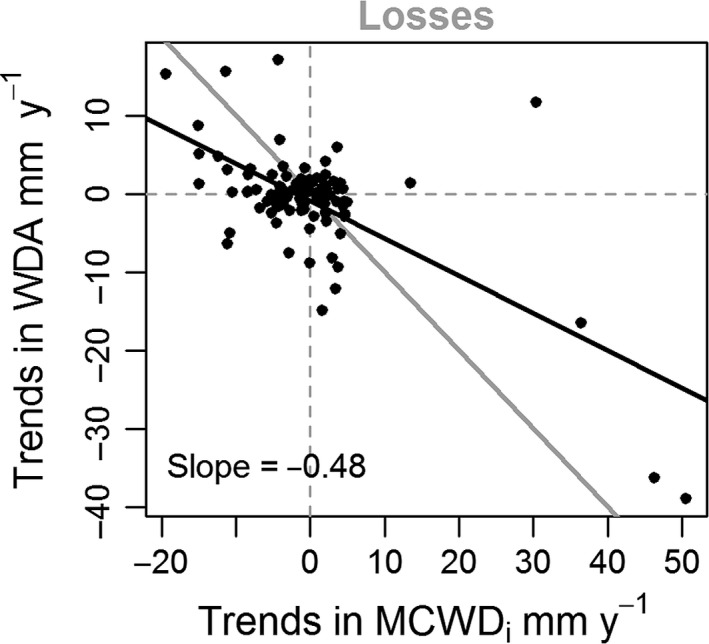
Relationship between trends in climate and functional composition of basal area mortality. The *y*‐axis represents plot trends in water deficit affiliation (WDA) per year calculated as the linear slopes of basal area‐based community weighted mean within the losses and *x*‐axis shows the trends in most extreme dry season within a census interval (MCWD_i_). The black line represents OLS linear regression, and in the 1:1 line is shown in grey. Note that changes in the tree community are correlated to changes in climate (Kendall *τ* = −0.2; *p* < 0.01), so that stronger drying trends favour the mortality of wet‐affiliated taxa. Correlations hold when outliers are removed (Kendall *τ* = −0.4; *p* < 0.05 when excluding outliers where climate trend >5 mm/year and trends in losses >10 mm/year) [Colour figure can be viewed at wileyonlinelibrary.com]

The trends in the intensity of extreme dry events (MCWD_i_) within each plot were significantly correlated with trends in the losses of community basal‐area WDA (Figure 4) and the fluxes of basal area WDA (Supporting Information Appendix S9). This indicates that the mortality of large wet‐affiliated trees has increased in plots where the dry seasons have become more extreme. Trends in climate were also negatively correlated to losses of stem‐based WD (Supporting Information Appendix S9). There was no significant correlation between trends in climate and trends in community weighted mean of any other trait or segments of the tree community (Supporting Information Appendix S9).

### 3.4 Floristic trends

The floristic changes represented by the linear trends in abundance for individual genera are generally consistent with the functional changes observed. There have been notable increases in the relative abundance of the dry‐affiliated genera *Bertholletia*,* Brosimum* and *Pseudolmedia*, together with sharp decreases for the wet‐affiliated *Aparisthmium*,* Fusaea, Inga* and *Mezilaurus*. Some large‐statured genera have increased significantly, such as *Mora, Couratari* and *Eschweilera*. A decrease in smaller‐statured taxa, such as *Ouratea, Aniba, Marlierea* and *Astrocaryum* is also observed (Supporting Information Appendix S12). Palms (Arecaceae) have significantly declined in abundance (Supporting Information Appendix S12) overall, with marked declines of *Oenocarpus* and *Astrocaryum*, even though the hyperdominant palm genus *Euterpe* has significantly increased in abundance across the basin.

**Figure 5:**
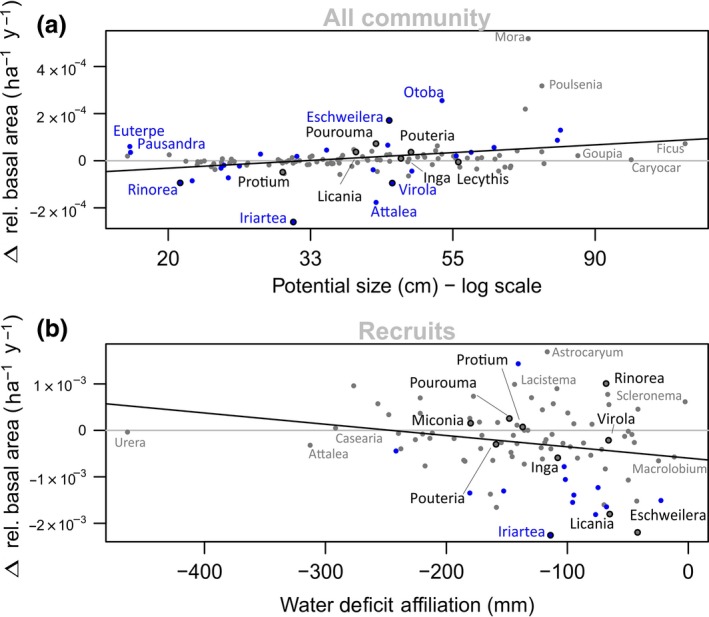
Floristic changes behind the detected functional shifts. The *y*‐axes represent mean linear slopes of plot level genera relative abundance across the Amazon in terms of number of individuals or basal area as a function of time, with each plots’ contribution weighted by the square root of plot area and monitoring period. Grey horizontal lines indicate zero change. The x‐axes represent genus‐level traits. (a) Trends in relative basal area within the whole community versus potential size (cm), plotted in the log scale to facilitate visualization; (b) trends in stem relative abundance within the recruits versus water deficit affiliation (mm). Genera that show significant trends in abundance across the basin are shown in blue. Black contour marks the 10 most abundant genera in terms of number of stems

By analysing abundance trends of different taxa, it is possible to identify which genera contribute the most to the observed overall changes in functional composition (i.e. PS basal area for the whole community and WDA stem density for recruits). The correlation between taxa PS and their trends in basal area was significant (Kendall *τ* = 0.2; *p*‐value < 0.01) and driven by an increase in some emergent and canopy genera such as *Eschweilera*,* Licania* and *Pouteria,* and a decrease in some sub‐canopy and understorey genera such as *Iriartea*,* Rinorea* and *Oenocarpus* (Figure 5 Supporting Information Appendix S12). The decrease in WDA within the recruits was also explained by changes in floristic composition across the Amazon (Kendall *τ* = −0.16; *p* < 0.05), with declines in the recruitment of new stems of wet affiliated Amazon genera such as *Mabea, Sterculia, Swartzia, Iryanthera* and *Theobroma* and recruitment increases for dry affiliates such as *Trema, Simarouba* and *Hieronyma* (Supporting Information Appendix S12).

Our analyses of the trends in abundance for the functional types defined for Amazonian trees by Fyllas et al. (2012) show small‐statured nonpioneer taxa to be significantly decreasing in abundance by 0.29% per year (Supporting Information Appendix S10). No significant trend was found for the other functional types (small‐statured pioneers, tall pioneers and tall nonpioneers). Note that this analysis was limited to the species classified by Fyllas et al. (2012) within these four functional types, which represent ca. 28% of the total number of individuals within our plots.

## 4 DISCUSSION

Here we report the first Amazon‐wide analyses of temporal trends in functional and floristic composition of lowland tree communities across 106 Amazonian inventory plots analysed over three decades. We find evidence of climate‐induced shifts in community compositional fluxes. Recruits have become more dry‐affiliated (Table 1) and the mortality of wet‐affiliated trees has increased in the areas where the drying trend was stronger (Figure 4), suggesting a direct effect of climate change on functional composition. This compositional shift is consistent with the detected intensification of the seasonal drought across the majority of permanent monitoring plots in Amazonia. The trends in potential size (PS) and wood density (WD) further indicate that changes within the Amazon forest are likely to be a consequence of a combination of drivers. However, our data also highlight the relative inertia of tropical forest communities in their ability to respond to environmental changes. For instance, the detected change in tree community fluxes, with recruits becoming more dry‐affiliated by 0.45 mm/year, is substantially smaller in magnitude than the actual climate trend of 1.1 mm/year.

The functional shifts observed here are mirrored by underlying floristic changes when our data are analysed in terms of discrete taxonomic units (Figure 5). The genus‐level floristic shifts behind the significant changes detected (Tables 1 and 2) illustrates how functional responses result from actual floristic change across Amazonian communities. However, the relationships between traits and floristic shifts also show significant scatter, suggesting idiosyncratic responses of each taxon and the complexity of this highly diverse system.

**Table 2:** Mean linear slopes in basal area‐based functional composition in Amazonia

Community	Potential size (cm/year)	Water deficit affiliation (mm/year)	Wood density (g cm^−3^ year^−1^)
All community	**0.03 (0.02**|**0.05)**	0.01 (−0.04|0.07)	3 × 10^−5^ (−6 × 10^−5^|1 × 10^−4^)
Gains (basal area)	**0.06 (0.01**|**0.12)**	−0.09 (−0.53|0.2)	−5 × 10^−4^ (−1 × 10^−3^|1 × 10^−4^)
Gains (recruits)	0.06 (−0.09|0.19)	−0.08 (−0.7|0.6)	−2 × 10^−4^ (−2 × 10^−3^|2 × 10^−3^)
Losses	0.13 (−0.08|0.33)	−0.33 (−1.2|0.5)	−1 × 10^−3^ (−2 × 10^−3^|3 × 10^−4^)
Net fluxes	−0.05 (−0.27|0.2)	0.24 (−0.7|1.19)	9 × 10^−4^ (−4 × 10^−4^|2 × 10^−3^)

*Note*

As in Table 1 but showing the results for basal area, see Figure 1 for details.

### 4.1 Climate‐induced changes in floristic composition

We detected an increase in the abundance of drought‐tolerant genera across Amazonia when analysing the recruitment and mortality within tree communities (Table 1, Figure 3), consistent with the hypothesis that tree communities are responding to the changes in climate. Our large‐scale results are consistent with tree community shifts towards more drought‐tolerant taxa reported in some neotropical (Butt et al., 2014; Enquist & Enquist, 2011; Feeley, Silman, et al., 2011) and west African forest localities (Fauset et al., 2012), and some temperate localities (McIntyre et al., 2015). Across Amazonia we find that greater mortality of wet‐affiliated taxa over time is related to the degree to which water stress has increased within each community, providing direct evidence of the influence of climate on community dynamics (Figure 4). This only emerged when analysing trends in basal area, indicating that it is driven by the increase in mortality of large wet‐affiliated trees. Indeed, large trees are expected to suffer the most under drought conditions by being more vulnerable to embolism, and thus more likely to die from hydraulic failure under drought stress (McDowell & Allen, 2015; Rowland et al., 2015). In addition, further supporting the conclusion that this reflects concerted, widespread changes in Amazon forest mortality, we also found an increase in the potential size of the dead trees when analyses were repeated considering each cluster of neighbouring plots as a single sample unit (Supporting Information Appendix S7). Somewhat surprisingly we find losses in stem‐based WD to be negatively correlated to changes in climate (Supporting Information Appendix S9), which suggests that WD may not be a good proxy of drought vulnerability.

Our results also suggest that other drivers are causing compositional shifts in Amazon forests. For example, although we did observe an increase in mortality of large, wet affiliated trees, consistent with the effect of the 2005 drought (Phillips et al., 2009), our analyses also show a slight increase in the basal area of potentially large tree genera across the basin. These results contradict the expectations that droughts would preferably impact larger trees but are in line with the suggestion that smaller‐statured trees are more vulnerable to droughts as they have shallower roots than larger‐statured trees (Condit et al., 1996; Fauset et al., 2012; Wright, 1992). Most likely, it appears that the increase in mortality of large trees in some areas of the basin (Figure 4, Supporting Information Appendix S9) is a consequence of drought events such as the 2005 drought (cf. Bennett et al., 2015; Phillips et al., 2010), but that it this mortality has been insufficient to counter a longer‐term tendency towards increased basal area of large‐statured taxa across Amazonia.

### 4.2 Additional drivers of compositional change

Larger trees have greater competitive capacity and are anticipated to gain disproportionately with additional resources (Coomes et al., 2011; Enquist, West, & Brown, 2009; Enquist, West, Charnov, & Brown, 1999). The increase in atmospheric CO_2_ potentially provides a parsimonious explanation for the observed relative increase in large‐tree genera (Table 2). If the increase in atmospheric CO_2_ of ca. 5% per decade since the 1980s (Conway & Tans, 2016) is stimulating plant growth or increasing water‐use efficiency, then taxa that tend to compete better for light, notably larger trees (Poorter et al., 2005), are likely to gain a greater competitive advantage (Falster & Westoby, 2003). Although further investigation is needed to confirm this hypothesis, our results show an increase in basal area of large‐statured taxa (Figure 5; Table 2) and a decrease in abundance of small‐statured taxa (Supporting Information Appendix S10), both consistent with the expectations from increased competition. Additionally, the relative increase of larger genera is also observed in the broader dataset including the fringes of Amazonia (*Extended Amazonia* see Supplementary material), which supports the inference of a ubiquitous driver of compositional change. Our observations are also in line with a series of stand‐level studies that show increases in above‐ground biomass, growth, mortality and recruitment across Amazonia—all trends expected as an outcome from increased atmospheric CO_2_ (Brienen et al., 2015; Phillips & Gentry, 1994; Phillips et al., 1998).

While considerable interest has focussed on the atmospheric and climatic changes, particularly drought, other environmental changes may be important. Conceivably one or more of these could have pervasive impacts on mature forests across the basin to rival or exceed any climate impacts. Amazonian forests have been hypothesized to be recovering from previous disturbances that are either natural (Chambers et al., 2013; Van Der Sande et al., 2016) or human‐driven, particularly in pre‐Colombian times (McMichael, Matthews‐Bird, Farfan‐Rios, & Feeley, 2017). If this is the case, we would expect the forest to be following a successional trajectory characterized by a shift from pioneers (low wood density) to mature forest species (high wood density; e.g. Chave et al., 2008; Connell & Slatyer, 1977; Lewis, Lloyd, et al., 2009). While the observed relative increase in basal area of larger taxa is consistent with widespread recovery from disturbance (Chave et al., 2008; Wright, 2005), the 106 Amazonian inventory plots show no significant shift in wood density (WD) across the whole community, or perhaps more importantly, among the recruits (Tables 1 and 2). We note that our WD data (Chave et al., 2009; Zanne et al., 2009) provide a less than perfect insight into actual change given that there is likely to be additional spatial and intra‐generic variation (Baraloto et al., 2010; Patiño et al., 2009) that we cannot account for. However, WD is considerably conserved across the phylogeny, and genus‐level wood density has been found to be adequate to distinguish between late successional and pioneer genera (Coelho de Souza et al., 2016). Moreover, the only functional group that has clearly *lost* ground over our monitoring window are the short‐statured nonpioneers—the best suited trees to late‐successional environments (Supporting Information Appendix S10). Compositional analyses further suggest that some pioneers are increasing, most notably an increase of 3.4% ha^−1 ^year^−1^ in the relative abundance of *Cecropia*, a key early successional taxon (Supporting Information Appendix S12), which typically dominates in early stages of succession but declines at later stages of the successional trajectory (Zalamea et al., 2012). The increase in the abundance of light‐demanding taxa may be a consequence of an acceleration in the canopy gap dynamics caused by the increase in baseline mortality rates (Baker et al., 2016; Brienen et al., 2015). Finally, if these forest plots are recovering from the impact of Amazonian peoples who favoured especially useful species, then we might expect domesticated taxa sensu Levis et al. (2017) to now be decreasing in abundance following the relaxation of this anthropogenic influence. No such trend is observed in our data (Supporting Information Appendix S11).

There has been considerable concern regarding the ecosystem impacts of widespread removal of large‐bodied frugivores. In particular, it has been repeatedly suggested that hunting will or may have already altered tree composition in tropical forests (e.g. Doughty et al., 2016; Osuri et al., 2016; Peres, Emilio, Schietti, Desmoulière, & Levi, 2016; Peres & Palacios, 2007; Terborgh et al., 2008) via dispersal failure of zoochoric trees (Chapman & Chapman, 1995). These tend to have high wood density and large stature, so a recruitment failure is predicted to lead to a reduction in the prevalence of both of these traits and thus in Amazonian biomass (Bello et al., 2015; Peres et al., 2016). This study was designed to understand floristic dynamics in intact Amazonian forests and not to evaluate the effects of hunting pressure, which is likely to more strongly affect areas adjacent to rural communities, roads and rivers (Peres & Lake, 2003). However, the increase in potential size and the lack of change in wood density within the recruits (Figure 3) contradict expectations of the hunting hypothesis as a driver of recent community composition change in intact forests, which, again, does not rule out the possibility of hunting pressure causing shifts in composition in particular locations where this activity is stronger.

### 4.3 The pace of change

Changes in tree communities are expected to substantially lag behind environmental changes as trees are sessile and long‐lived (Blonder et al., 2017; Davis, 1989; Hubbell, 2001; Lenoir, Gegout, Marquet, De Ruffray, & Brisse, 2008; Svenning & Sandel, 2013). Our results are consistent with this prediction. In other systems where climate gradients are almost unidirectional, it is possible to assess the speed at which communities are expected to be moving across spaces to follow climate (Devictor et al., 2012), but this is not the case for precipitation in Amazonia where precipitation regime is heterogeneous at multiple spatial scales. However, by ensuring that climate and water‐deficit affiliation are calculated in the same scale we can compare the degree to which climate and communities are changing. Across Amazonia, plots have undergone an average drying trend of −1.1 mm/year MCWD (Figure 2). Notably, change in tree communities did not keep pace with the change in climate—the mean trend in water‐deficit affiliation for the whole community is two orders of magnitude smaller (0.01 mm/year, Table 1), with confidence intervals overlapping zero. However, a much shorter lag is observed when analysing the net fluxes, that is recruits−deaths (−0.45 mm/year, Table 1), indicating that although responses are slow, this system has some dynamic capacity to respond to changes in climate.

The observed pace of change is a reflection of the nature of these communities. Recruitment and mortality of trees ≥10 cm D averaged nine trees per hectare per year between 1985 and 2010 across the basin (Brienen et al., 2015). Considering that in our data mean stem density is 520 trees ha^−1^ and mean plot‐monitoring length is 14 years, we can expect by the final census an accumulated turnover of ≈24% of stems. Thus, we should expect a priori that whole community‐level composition would not only be affected by changes over the measurement period but would also reflect legacy effects of recruitment and mortality processes occurring decades prior to the onset of the monitoring period (Davis, 1989). Our results provide empirical evidence of the inertia within this system and clearly raise concerns about whether forests here will be able to track further climate change anticipated over coming decades.

This study provides the first Amazon‐wide picture of functional and floristic dynamics over the last 30 years. Models have predicted a strengthening of the dry season over the Amazon (Boisier, Ciais, Ducharne, & Guimberteau, 2015; Joetzjer, Douville, Delire, & Ciais, 2013), and an increase in water‐stress as a consequence of rising temperature (Pokhrel, Fan, & Miguez‐Macho, 2014). But there have been few attempts to account for changes in composition, which may modulate the whole forest ecological impact of ecophysiological drivers such as increasing vapour pressure deficit (Levine et al., 2016; Sakschewski et al., 2016). The velocity and the magnitude of compositional changes presented here should be considered in attempts to model the dynamics of these forests under future climate. Our results show that a slow shift towards a more dry‐affiliated Amazonia is underway. If such a floristic shift is substantial enough to increase the resilience of Amazon tree communities to future droughts, it will still come with a price in terms of tree biodiversity, as wet‐affiliated restricted taxa represent the majority of Amazonian tree flora (Esquivel‐Muelbert, Baker, et al., 2017). Furthermore, although our results demonstrate that changes in composition are possible, the inertia intrinsic to these communities means that they will substantially lag behind climate change. Droughts are continuing to impact the basin (Erfanian et al., 2017; Jiménez‐Muñoz et al., 2016). If this lag persists, intact Amazonian forests may be increasingly dominated by sub‐optimally adapted trees in terms of their preferred climate space, potentially threatening the ability of these ecosystems to provide key services such as protecting biodiversity and sequestering and storing carbon.

## AUTHOR CONTRIBUTIONS

AE‐M, TRB, SLL and OLP designed the study. AE‐M carried out the analyses with inputs from TRB, KGD, SLL and OLP. AE‐M and OLP wrote the manuscript with input from TRB, KGD and SLL. OLP, JL and YM conceived the RAINFOR forest plot network; EG, TRB, GL‐G and GCP contributed to its development. OLP, RJWB, TRF, TRB, AM‐M, LA, EA, BSM, B‐HM, NH, MS, EV, JC, EG and YM coordinated data collection with the help of many co‐authors. All co‐authors collected field data and commented on the manuscript.
