# Supplementary material for: Compositional response of Amazon forests to climate change
Source: Glob Chang Biol. 2018 Nov 8;25(1):39–56. doi: 10.1111/gcb.14413 (PMC6334637; doi:10.1111/gcb.14413)
Supplement: Supplementary file 1 [file GCB-25-39-s001.pdf]

Supplementary information

Appendix S1 –Amazonia inventory data

Our intention was to investigate the effects of increases in water stress within the moist Amazonian forests (mean annual MCWD > -300 mm y^{-1}) that have experienced the 2005 and 2010 droughts. This *Core Amazonia* dataset included 106 plots across Amazonia (Figure S1, green circles). We also repeated the analyses using a more inclusive dataset, which we call *Extended Amazonia.* It consists of 165 plots, including those analysed here plus plots in the border of the Amazon where mean MCWD is more negative than -300 mm y^{-1} and those which were not monitored throughout 2005 and 2010. The results for the Extended Amazonia are similar to those found for the most restricted data (Appendix S5 and S6). However, in line with the expectations that moist forests would be more affected by shifts in climate, Extended Amazonia shows a less notable climate signal. The results using Extended Amazonia are omitted from the main text and are provided in Supplementary information.

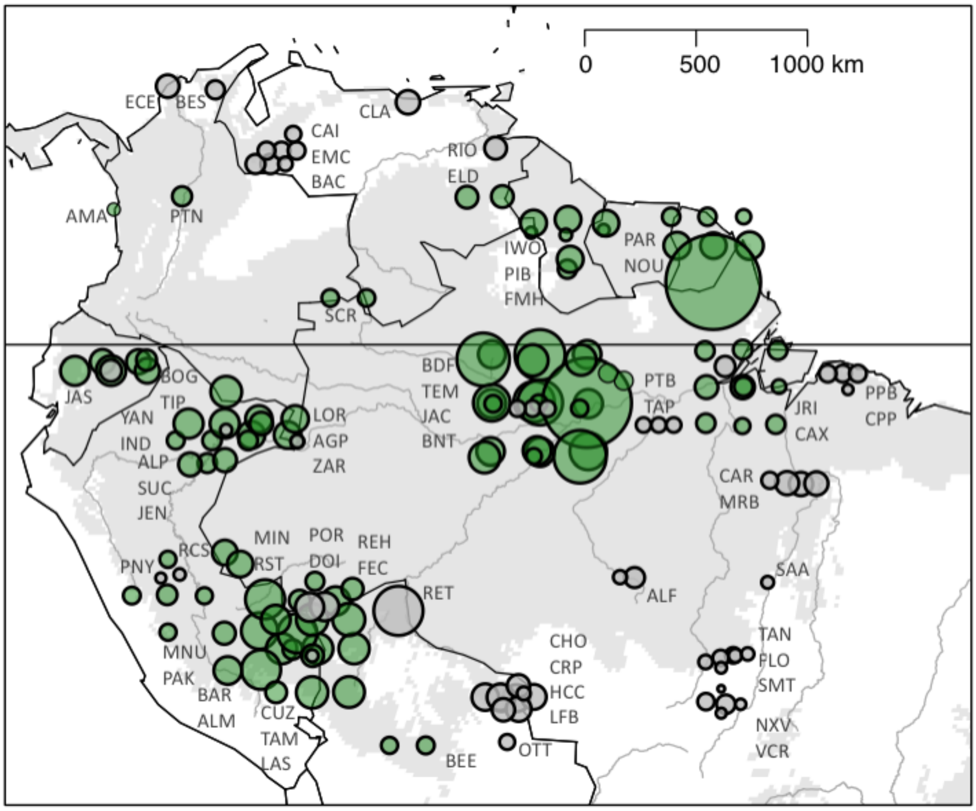

Figure S1 – Sample effort in the analyses. Circles represent 165 tree inventory plots across the Amazon used in this study. In green the 106 inventory plots used for the analyses in the main text (*Core Amazonia*) - tropical moist forests (mean MCWD > -300 mm y^{-1}) that have been monitored during the 2005 and 2010 droughts. The area of each circle represents the sample effort per plot as square root of plot area * monitoring period. The locations of overlapping plots have been adjusted to allow visibility. Three letter codes represent a cluster of nearby plots.

Appendix S2 - Independent Amazonian tree traits

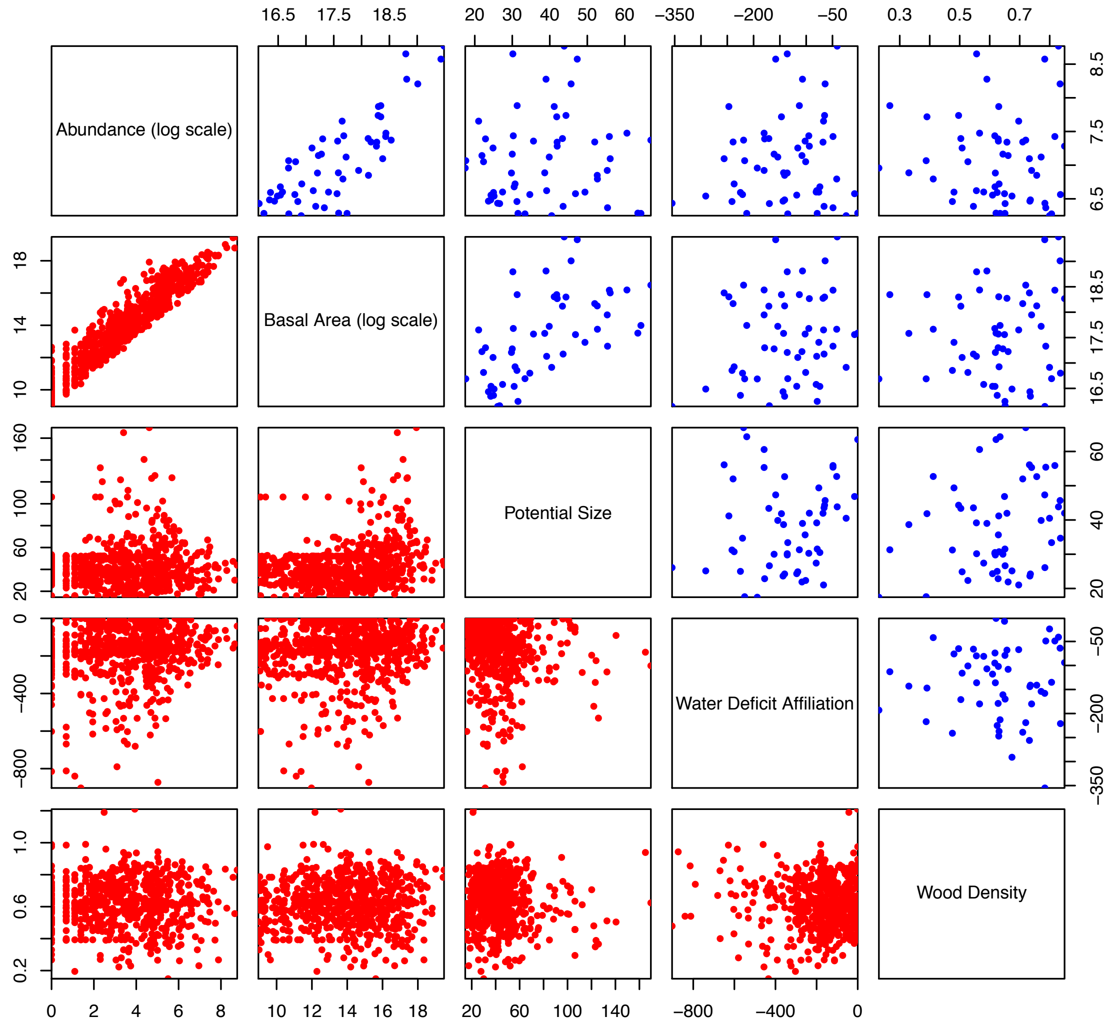

Figure S2 - Relationship between genus-level traits and dominance. In red on the lower panels graphs show relationship including 679 genera from *Extended Amazonia* dataset. Top graphs, in blue, show the relationships for the 57 genera with more than 500 individuals across the plots analysed here. Abundance and basal area are presented in the natural logarithm scale. Water deficit affiliation values were calculated in Esquivel-Muelbert *et al.* (2017), wood density was obtained from the Wood Density Database (Chave *et al.*, 2009, Zanne *et al.*, 2010), and potential size from Fauset *et al.* (2015).

Appendix S3 Trends in climatological water deficit across Amazonian plots

Maximum cumulative water deficit (MCWD) is a metric calculated from precipitation and evapotranspiration data (Aragao *et al.*, 2007, Chave *et al.*, 2014). There are two major sources of information of precipitation for the Amazon Basin: ground-based data from weather stations and satellite-based data from the Tropical Rainfall Measuring Mission (TRMM - Huffman *et al.*, 2007). Data from weather stations are interpolated at 0.5° resolution by the climatological research unit (CRU - Harris *et al.*, 2014) are available from 1901 to 2014. Ground-based data are available for a longer period of time, however the number of weather stations within the Amazon can compromise the quality of these data. Thereby, we verify the plot-level yearly values of MCWD calculated using only CRU data against MCWD using precipitation data from TRMM.

For evapotranspiration we used CRU data, which is calculated using the Penman–Monteith equation (Allen *et al.*, 1994). To understand the whole of changes in precipitation vs. temporal variation in evapotranspiration on the observed changes in MCWD we calculate MCWD trends maintaining evapotranspiration constant at 100 mm. For these examinations of the components of MCWD we used the plots from the *Extended Amazonia* dataset.

Results

MCWD values based on CRU and TRMM tend to agree overall ($R^2 = 0.8$, $P<0.0001$) (Figure S3.1). When comparing the overall slope from 1985 for the basin from bootstrapped mean and 95% CI of individual slopes, both CRU and TRMM show a decrease in MCWD, with TRMM showing slight higher values: CRU -1.12 (95% CI -1.3; -0.9) and TRMM -1.3 (95% CI -1.8; -0.7).

Both datasets show differences on how trends in MCWD are distributed across space, especially for South East Amazon (Figure S3.2), which is not included in the main analyses, i.e. *Core Amazonia*. Overall CRU-based MCWD showed similar results from TRMM-based values and the major difference in the direction of trends for some plots seems to be related to the time window analysed: when the same time window is analysed (1998-2013) both datasets show very similar trends for the basin: TRMM -1.2 (95% CI -2; -1); CRU -0.8 (-1.1; -0.5). Thereby, we used CRU-data in the analyses, as it spans over the same time window as the vegetation dataset.

Values of trends in MCWD for constant evapotranspiration (-0.7 mm y^{-1}, 95% CI = -0.9, -0.5; results for Core Amazonia) did differ marginally significantly from trends when allowing

evapotranspiration to vary (-1.1 mm y^{-1}; 95% CI = -1.3, -0.9). This different reflects the shift in both temperature and precipitation across the Amazon over the last 30 years.

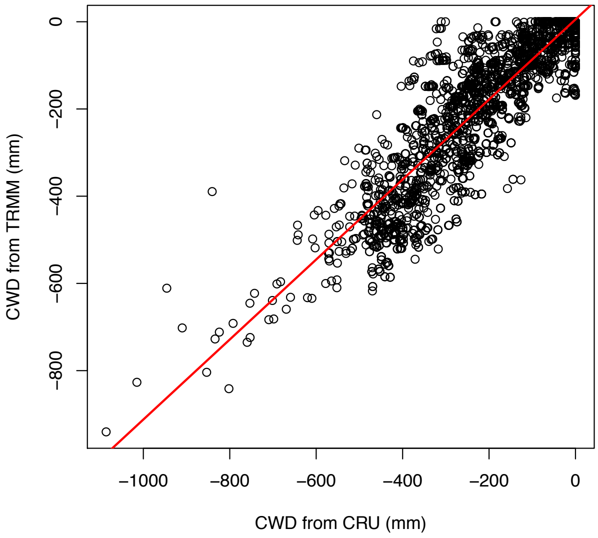

Figure S3.1 – Relationship between CRU and TRMM-based MCWD values. Black dots show annually MCWD values between 1998 and 2013 for each of the 165 plots analysed here. Red line represents a linear regression ($R^2 = 0.8$, $P < 0.0001$).

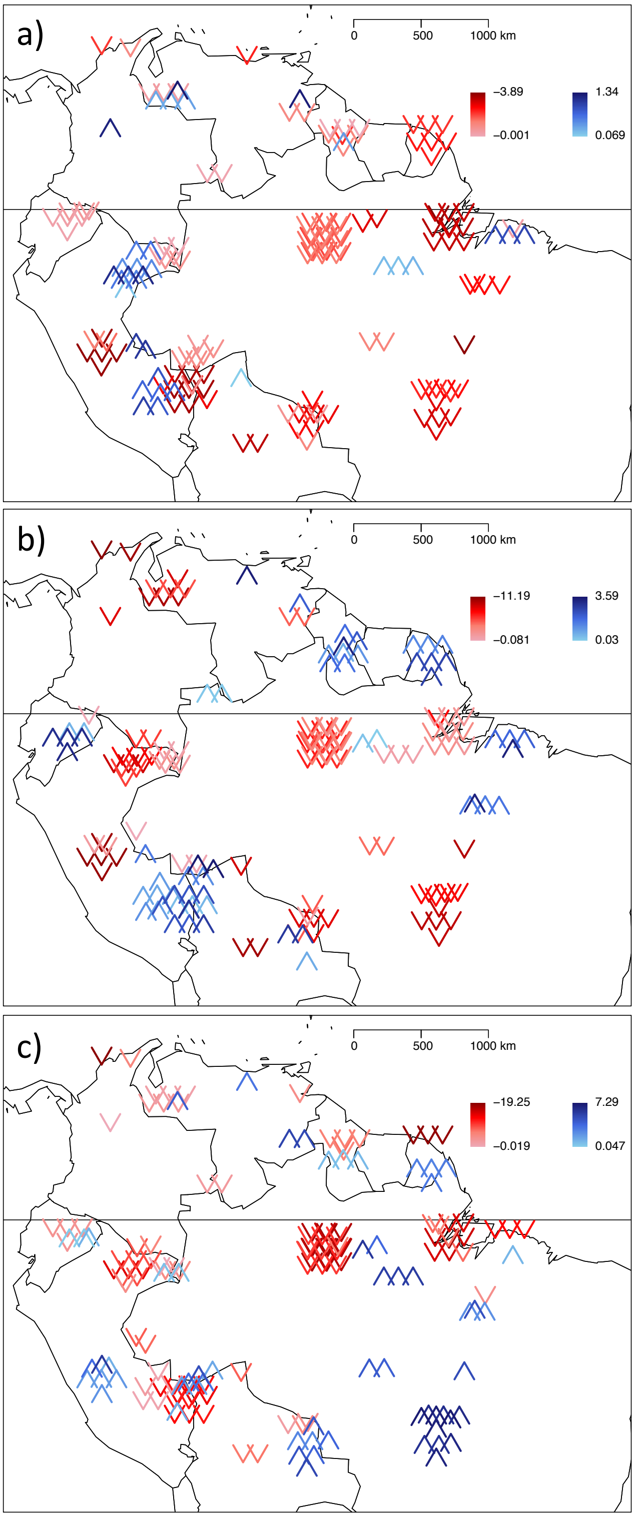

Figure S3.2 – Spatial pattern of MCWD across the plots analysed here using different datasets. (a) CRU-based MCWD from 1985; (b) from 1998 and (c) TRMM-based MCWD from 1998. The direction and colour of the arrows represent the direction of the trend (red, negative and blue positive), whilst the intensity of the colour shows the trend's intensity. Note that the scale of the trend is similar for TRMM and CRU from 1998 and the direction of the trend varies mostly in South East Amazonia, which is in not included in the analyses in the main text.

Appendix S4 – Determining appropriate weights for individual plot functional and floristic trends.

Plot area and monitoring period are expected to affect the plot-level trends as forest stands monitored over shorter periods or smaller area are more likely to be affected by stochastic phenomena, such as tree fall. Thus, monitoring plots with greater sample effort should represent better the trends in functional and floristic composition. When analysing a combination of inventory plots with different sample sizes and monitoring periods, smaller plots and those monitored over a shorter length of time should bring more variation to the overall trend increasing the error to estimate ratio. In these cases, greater weights are given to plots with greater sample effort (Brienen *et al.*, 2015, Lewis *et al.*, 2009).

In order to decide which are the best weights to be implemented in our analyses we evaluate the effect of monitoring period and plot area on the deviation from the mean estimate for each model, i.e. the absolute difference between the change in functional composition in each plot and the Pan-Amazonian mean slope for each model. The effect of sample effort was assessed through the slope of the relationship between deviation from mean estimate and area or monitoring period (Figure S4.1a and b) and compared to the effect when weights are applied (Figure S4.1 c- f). To verify whether the weighting procedure is appropriate we tested for the relationship between weights and residuals vs. weights (Figure S4.2).

When including the weights in the models their fit improved overall for our main analyses, the bootstrap mean of slopes (Figure S4.1). The relationship between residuals and area or monitoring period flatten when using the squared root of these parameters as weights for each plot. Following this exploratory analysis the squared root of area * squared root of monitoring period seems to be an adequate weighting procedure for this type of analyses (Figure S4.2). The results differ for the LMM analyses where weights seem not to be necessary (Figure S4.3). Even light weights, such as the cubic root of area and monitoring period seem to overweight these parameters (Figure S4.3 b and c). This indicates that the random effect including in the model seem to be enough to account for the variation among plots in terms of area and monitoring period. Thus, no weights were applied for LMM analyses.

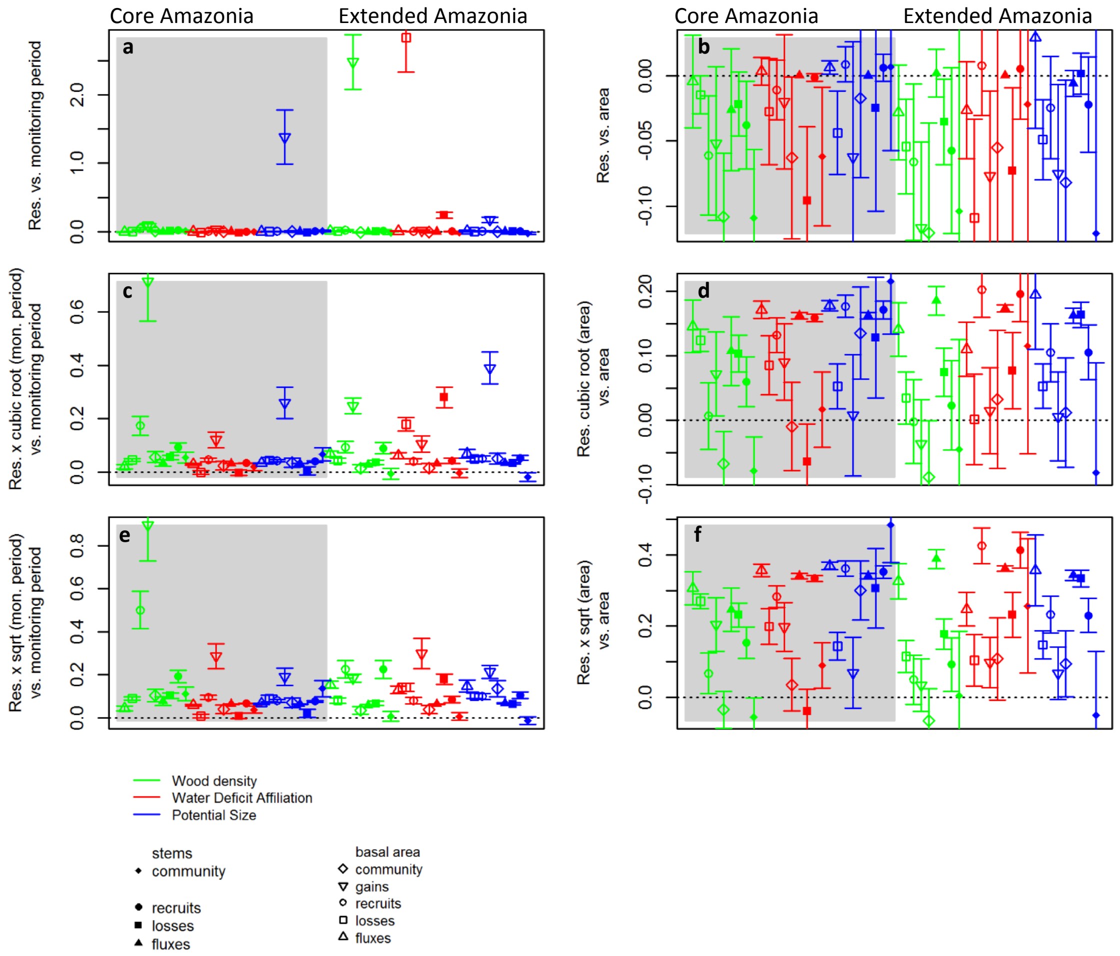

Figure S4.1. Determining appropriate weights for individual plot functional change. Linear slopes (dots) and error bars of the relationship between residuals of each model versus plot monitoring period (a) and area (b). Slopes are expected to overlap zero when there is no influences of sample effort on the variation of the estimate. Weighting all plots by squared root of monitoring period (e) and area (f) shows to be the most effective procedure – most slopes overlapping zero – when compared to the cubic root of monitoring period (c) and area (d) and. (g) shows that stronger weights – monitoring period – would be to strong.

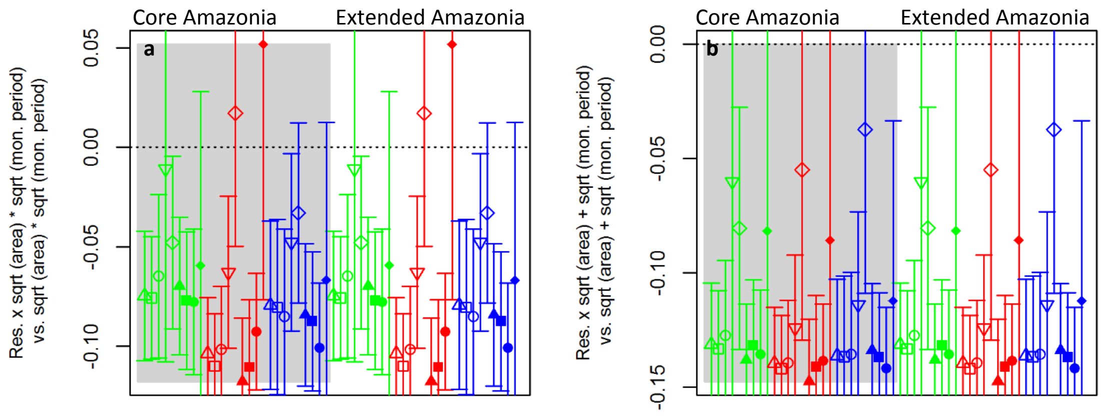

Figure S4.2. Combining weights for monitoring period and area. Squared root of area * the squared root of monitoring period (a) are more adequate than Squared root of area + the squared root of monitoring period, as slopes are closer to zero in (a).

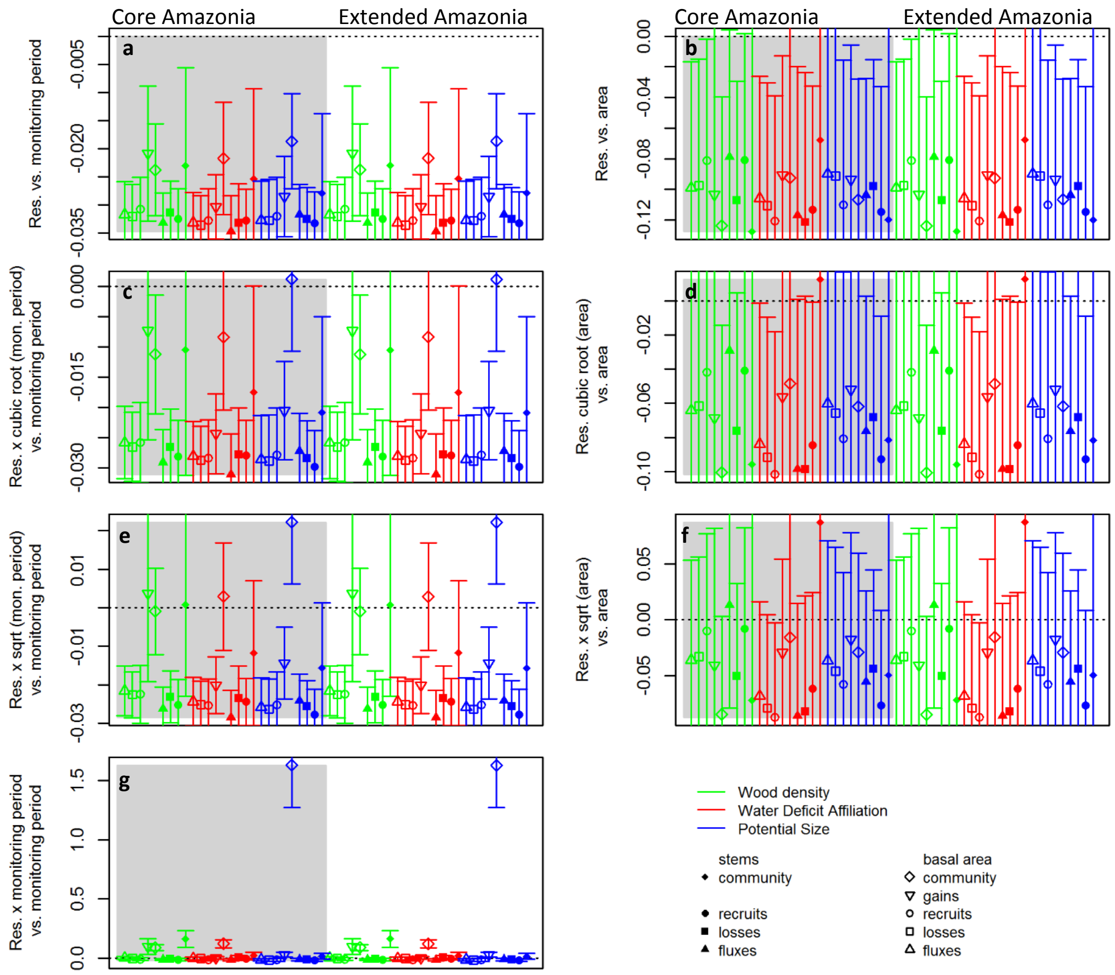

Figure S4.3. As Figure S4.1 but for residuals of GLMM models. Note that weights are not necessary in this case as most slopes overlap zero.

Appendix S5 - Trends in functional composition for Extended Amazonia

Table S5.1 – Mean linear slopes in individual-based functional composition across the 165 plots in the Extended Amazon dataset. For each trait, the bootstrap mean annual changes in community weighted mean (CWM) weighted by the squared root of plot size x monitoring period. In brackets: 95% confidence intervals. CWM was calculated for: water deficit affiliation (WDA), potential size (PS) and wood density (WD). The analyses were repeated for recruits, dead trees and the difference between recruits and dead trees (net fluxes). In bold significant trends, i.e. where CIs do not overlap zero.

Community	Potential size ($cm\ y^{-1}$)	Water Deficit affiliation ($mm\ y^{-1}$)	Wood Density ($g\ cm^{-3}\ y^{-1}$)
Whole community	-0.001 (-0.03|0.02)	-0.12 (-0.30|0.01)	$-2x10^{-4}$ ($-6x10^{-4}$ | $8x10^{-5}$)
Gains (recruits)	0.09 (-0.04|0.22)	-0.48 (-1|0.04)	$3x10^{-4}$ ($-9x10^{-4}$ | $2x10^{-3}$)
Losses	-0.04 (-0.16|0.07)	-0.21 (-1.03|0.5)	$-5x10^{-4}$ ($-2x10^{-3}$ | $6x10^{-4}$)
Net fluxes	0.13 (-0.04|0.3)	-0.35 (-1.35|0.68)	$8x10^{-4}$ ($-7x10^{-4}$ | $2x10^{-3}$)

Table S5.2 – Basal area-based annual plot level trends in functional composition across the Amazon Basin. As table 5.1 but at for basal area-based analyses.

Community	Potential size ($cm\ y^{-1}$)	Water Deficit affiliation ($mm\ y^{-1}$)	Wood Density ($g\ cm^{-3}\ y^{-1}$)
All community	**0.03 (0.01|0.05)**	-0.06 (-0.15|0.02)	$-4x10^{-5}$($-2\ x10^{-4}$| $7x10^{-5}$)
Gains (basal area)	0.02 (-0.04|0.09)	-0.31 (-0.71|0.02)	**$-8x10^{-4}$ ($-2x10^{-3}$ | $-2x10^{-4}$)**
Gains (recruits)	0.10 (-0.07|0.27)	-0.22 (-0.87|0.46)	$8x10^{-4}$($-6x10^{-4}$| $2x10^{-3}$)
Losses	-0.17 (-0.5|0.10)	0.20 (-0.7|1.1)	$-1x10^{-3}$ ($-3x10^{-3}$| $6x10^{-5}$)
Net fluxes	0.24 (-0.05|0.6)	-0.49 (-1.53|0.49)	$1x10^{-3}$ ($-3x10^{-4}$| $2x10^{-3}$)

Appendix S6 - Results from the LMM

Table S6.1 – Annual plot level trends in functional composition across the Core Amazonia dataset. Intercept, slope, and percentage change per year (in brackets), of plot-level water deficit affiliation, potential size and wood density between 1985 and 2015. Trends were calculated by fitting a linear mixed model (LMM) to a time-series of census level community-weighted mean for each trait. The LMM consider plot slope and intercept as random effects. The analyses were repeated for recruits, losses and the difference between recruits and losses (net fluxes). Values in bold and followed by + represent slopes that significantly differ from zero, considering respectively $\alpha = 0.05$ and $\alpha = 0.1$.

		Abundance		Basal Area	
Trait	Community	Intercept	Slope (%)	Intercept	Slope (%)
Potential size	All community	42	0.01 (0.02)+	51	**0.03(0.06)**
(cm y^{-1})	Gains (growth)			50	0.03 (0.06)
	Gains (recruits)	39	0.06 (0.2)+	39	0.07 (0.2)+
	Losses	39	-0.01 (-0.03)	43	0.1 (0.2)+
	Net fluxes	-0.3	0.05 (16)	5	-0.04 (-0.7)
Water Deficit Affiliation	All community	-126	0.004 (0.003)	-135	0.01 (0.01)
(mm y^{-1})	Gains (growth)			-138	0.02 (0.04)
	Gains (recruits)	-125	-0.2 (-0.2)	-127	-0.22 (-0.18)
	Losses	-130	-0.02 (-0.02)	-132	-0.14 (-0.11)
	Net fluxes	6	-0.18 (-3.1)	-2	0.07 (3)
Wood Density	All community	0.64	$-8x10^{-6}$ (-0.001)	0.64	$3\ x10^{-5}$ (0.005)
(g cm^{-3} y^{-1})	Gains (growth)			0.63	$-2\ x10^{-4}$ (-0.03)+
	Gains (recruits)	0.62	$-5x10^{-4}$ (-0.08)+	0.61	$-4\ x10^{-4}$ (-0.08)
	Losses	0.62	$-3x10^{-4}$ (-0.04)	0.63	**$-1\ x10^{-3}$ (-0.16)**
	Net fluxes	-0.005	$7\ x10^{-5}$ (1.45)	-0.01	$6\ x10^{-4}$ (11)+

Table S6.2 – As table S6.1 but for Extended Amazonia dataset.

		Abundance		Basal Area	
Trait	Community	Intercept	Slope (%)	Intercept	Slope (%)
Potential size	All community	42	-0.001 (-0.002)	51	**0.03(0.05)**
(cm y^{-1})	Gains (growth)			51	0.02 (0.04)
	Gains (recruits)	39	0.06 (0.14)+	39	**0.06 (0.15)**
	Losses	40	-0.06 (-0.14)+	45	-0.03 (-0.06)
	Net fluxes	-1	**0.1 (8)**	4	-0.04 (-1.2)
Water Deficit Affiliation	All community	-149	-0.16 (-0.11)+	-156	-0.08 (-0.05)
(mm y^{-1})	Gains (growth)			-159	0.01 (0.01)
	Gains (recruits)	-144	**-0.42 (-0.29)**	-146	**-0.39 (-0.27)**
	Losses	-156	0.23 (0.15)	-160	0.18 (0.11)
	Net fluxes	8	-0.32 (-3.9)	5	-0.31 (-6)
Wood Density	All community	0.64	$-2x10^{-4}$ (-0.03)	0.64	$-3x10^{-5}$ (-0.01)
(g cm^{-3} y^{-1})	Gains (growth)			0.62	$-2x10^{-4}$ (-0.04)+
	Gains (recruits)	0.61	$-1x10^{-4}$ (-0.02)	0.60	$-1x10^{-4}$ (-0.02)
	Losses	0.61	$-3x10^{-4}$ (-0.05)	0.62	**$-8x10^{-4}$ (-0.13)**
	Net fluxes	-0.01	$4x10^{-4}$ (4.8)	-0.01	$6x10^{-4}$ (8)+

Appendix S7 – Tenting the influence of spatial autocorrelation

Table S7.1 - Mantel correlation between the trends in functional composition and the spatial distance (latitude and longitude) between our plots and groups of plots (cluster, three letter codes in figure S1). Values in bold represent Mantel correlations that significantly differ from zero, considering respectively α = 0.05 rejecting the null hypothesis of absence of spatial structure in our data. Note that the correlation between the spatial distance between the plots and the similarity between the trends is often low and is not significant when analysed are performed at the cluster level.

		Abundance		Basal area	
Trait	Community	Plot	Cluster	Plot	Cluster
Potential size ($cm\ y^{-1}$)	All community	0.04	-0.06	0.003	-0.10
	Gains (growth)			**0.1**	-0.05
	Gains (recruits)	**0.12**	-0.01	**0.09**	-0.03
	Losses	**0.10**	0.06	**0.07**	-0.06
	Net fluxes	**0.11**	-0.02	0.06+	-0.07
Water Deficit Affiliation ($mm\ y^{-1}$)	All community	-0.01	-0.15	0.04	-0.08
	Gains (growth)			0.04	-0.06
	Gains (recruits)	**0.07**	0.01	0.04	-0.07
	Losses	0.03	0.08	0.06+	0.02
	Net fluxes	**0.08**	0.05	0.05	-0.01
Wood Density ($g\ cm^{-3}\ y^{-1}$)	All community	0.01	-0.06	0.05+	-0.04
	Gains (growth)			0.05	-0.06
	Gains (recruits)	**0.07**	-0.06	0.06+	-0.07
	Losses	**0.07**	-0.08	**0.08**	-0.04
	Net fluxes	**0.09**	-0.06	**0.08**	-0.005

Table S7.2 – Mean linear slopes in individual-based functional composition across the 38 clusters in the Core Amazon subset. For each trait, the bootstrap mean annual changes in community weighted mean (CWM) weighted by the squared root of cluster area x mean monitoring period across plots of a cluster. In brackets: 95% confidence intervals. CWM was calculated for: water deficit affiliation (WDA), potential size (PS) and wood density (WD). The analyses were repeated for recruits, dead trees and the difference between recruits and dead trees (net fluxes). In bold significant trends, i.e. where CIs do not overlap zero. Note that the do not differ from the result at the plot-level analyses (Table 1 and 2, main text), however here we see an indication of increase mortality of large trees.

Community	Potential size ($cm\ y^{-1}$)	Water Deficit affiliation ($mm\ y^{-1}$)	Wood Density ($g\ cm^{-3}\ y^{-1}$)
Whole community	0.01 (-0.001|0.02)	-0.004 (-0.06|0.04)	$-8x10^{-6}$ ($-1x10^{-4}$ | $1x10^{-4}$)
Gains (recruits)	0.08 (-0.09|0.24)	**-0.57 (-1.15|-0.004)**	$2x10^{-4}$ ($-1x10^{-3}$ | $2x10^{-3}$)
Losses	**0.13 (0.002|0.3)**	-0.11 (-0.65|0.4)	$2x10^{-4}$ ($-1x10^{-3}$ | $2x10^{-3}$)
Net fluxes	-0.05 (-0.36|0.17)	-0.49 (-1.13|0.11)	$-3x10^{-5}$ ($-2x10^{-3}$| $2x10^{-3}$)

Table S7.3 – Basal area-based annual cluster level trends in functional composition across the Amazon Basin. As table 7.1 but at for basal area-based analyses.

Community	Potential size ($cm\ y^{-1}$)	Water Deficit affiliation ($mm\ y^{-1}$)	Wood Density ($g\ cm^{-3}\ y^{-1}$)
All community	**0.03 (0.01|0.04)**	-0.01 (-0.07|0.06)	$4x10^{-6}$($-1x10^{-4}$| $1x10^{-4}$)
Gains (basal area)	0.06 (-0.02|0.14)	-0.15 (-0.9|0.35)	$-7x10^{-4}$($-2x10^{-3}$| $3x10^{-4}$)
Gains (recruits)	0.05 (-0.21|0.25)	0.04 (-0.9|1.23)	$5x10^{-4}$($-2x10^{-3}$| $4x10^{-3}$)
Losses	0.1 (-0.16|0.34)	-0.4 (-1.6|0.8)	$-8x10^{-4}$ ($-2x10^{-3}$| $8x10^{-4}$)
Net fluxes	-0.02 (-0.31|0.28)	0.31 (-1.17|1.6)	$8x10^{-4}$ ($-6x10^{-4}$| $2x10^{-3}$)

Appendix S8 - Trends in functional composition using species-level traits

Table S8.1 – Mean linear slopes in stem-based functional composition across the Amazon based on species-level traits. The analysis was performed using trait information at the finer taxonomic level as possible. Missing data were filled using mean trait value of higher taxonomic groups as explained in the main text. For each trait we show the bootstrap mean annual changes in community weighted mean (CWM) and 95% confidence intervals (CI, in brackets) weighted by the product of the squared root of plot size and monitoring period. CWM is calculated using: water deficit affiliation (WDA), potential size (PS) and wood density (WD). Species-level trait data were used for 28%, 43% and 0.3% of the stems for PS, WDA and WD, respectively. The analyses were repeated for recruits, losses and the difference between recruits and dead trees (net fluxes). Significant trends are in bold, i.e. where 95% CIs do not overlap zero.

Community	Potential size (cm y^{-1})	Water Deficit affiliation (mm y^{-1})	Wood Density (g cm^{-3} y^{-1})
Whole community	0.005 (-0.002|0.01)	0.01 (-0.03|0.04)	$-1x10^{-5}$ ($-9x10^{-5}$ | $6x10^{-5}$)
Gains (recruits)	0.07 (-0.03|0.2)	**-0.45 (-1|-0.02)**	$-3x10^{-4}$ ($-2x10^{-3}$| $1x10^{-3}$)
Losses	0.1 (-0.006|0.2)	-0.1 (-0.6|0.3)	$2x10^{-4}$ ($-7x10^{-4}$| $1x10^{-3}$)
Net fluxes	-0.03 (-0.2|0.1)	-0.5 (-1|0.1)	$-7x10^{-4}$ ($-2x10^{-3}$| $8x10^{-4}$)

Table S8.2 – Mean linear slopes in basal area-based functional composition across the Amazon based on species-level traits. As Table S8.1 but showing the results for basal area, see Figure 1 for details.

Community	Potential size (cm y^{-1})	Water Deficit affiliation (mm y^{-1})	Wood Density (g cm^{-3} y^{-1})
All community	**0.03 (0.02|0.04)**	0.02 (-0.03|0.07)	$3x10^{-5}$($-6\ x10^{-5}$| $1x10^{-4}$)
Gains (basal area)	0.04 (-0.02|0.1)	0.02 (-0.4|0.3)	$-5x10^{-4}$($-1\ x10^{-3}$| $1x10^{-4}$)
Gains (recruits)	0.06 (-0.09|0.19)	-0.08 (-0.7|0.6)	$-2x10^{-6}$($-2\ x10^{-3}$| $2x10^{-3}$)
Losses	0.12 (-0.08|0.33)	-0.33 (-1.2|0.5)	$-1x10^{-3}$ ($-2x10^{-3}$| $3x10^{-4}$)
Net fluxes	-0.07 (-0.3|0.15)	0.34 (-0.6|1.3)	$9x10^{-4}$ ($-4x10^{-4}$| $2x10^{-3}$)

* Note that results from tables S8.1 and S8.2 are very similar to the results in Table 1 and 2 in the main text.

Appendix S9 - Relationship between trends in climate and functional composition

Table S9.1 Relationship between trends in climate and functional composition. Kendall's τ coefficient of correlation between linear slopes of community weighted mean (CWM) and the linear slopes in cumulative water deficit within each census interval (MCWDi). CWM trends are calculated for: potential size, water deficit affiliation and wood density. In bold correlations that differ from zero considering α=0.05 when testing the null hypothesis of no relationship between changes in climate and changes in functional composition. In brackets Kendall's τ coefficient of correlation when outliers in terms of climate trends (see figure 4 in main text). Note that the null hypothesis is rejected for basal area-based losses and fluxes of water deficit affiliation, meaning large wet affiliated trees tend to die more in areas where the trend for climate to become drier is stronger.

Trait	Community	Abundance	Basal area
Potential size	All community	-0.04 (-0.07)	0.0004(0.02)
	Gains (basal area)		0.03(0.03)
	Gains (recruits)	0.04(0.1)	0.01(0.05)
($cm\ y^{-1}$)	Losses	0.11(0.13)	0.04(0.04)
	Net fluxes	0.05(0.07)	-0.03(-0.04)
Water Deficit	All community	-0.03 (-0.01)	0.02 (0.04)
	Gains (basal area)		0.03 (0)
Affiliation	Gains (recruits)	0.07 (0.09)	0.07 (0.1)
($mm\ y^{-1}$)	Losses	-0.06 (-0.05)	**-0.20 (-0.17)**
	Net fluxes	0.10 (0.09)	**0.18 (0.15)**
Wood Density	All community	-0.07(-0.05)	-0.08(-0.06)
	Gains (basal area)		-0.05(-0.09)
	Gains (recruits)	-0.03(-0.02)	0.02(0.03)
($g\ cm^{-3}\ y^{-1}$)	Losses	**-0.14(-0.15)**	-0.01(0.005)
	Net fluxes	0.03(0.05)	-0.03(-0.04)

Appendix S10 – Trends of Amazonian plant functional types

We investigate the trend in abundance for the four Amazonian functional types defined by Fyllas *et al.* (2012). This approach is an independent test for our hypotheses as it integrates a series of foliar traits not used in our core analyses. Fyllas *et al.* (2012) classified 276 Amazonian tree species within four functional types based on foliar and structural data: small pioneer, small statured non-pioneer, tall pioneer and tall non-pioneers, see Fyllas *et al.* (2012) for details on each functional group and how they are defined. We applied this classification to the 243 species described by Fyllas *et al.* (2012) that were also found in our data and test for the trend in abundance within each of these functional types. Then we calculated the trend in abundance of each functional type following the same procedure described in the main text for individual taxa.

Table S10 - Annual plot level trends for functional types *sensu* Fyllas *et al.* (2012) across the Core Amazonia dataset. Intercept, slope, and percentage change per year in abundance for each of the four functional types and all the remaining taxa grouped together (other taxa) between 1985 and 2015. Trends were calculated by fitting a linear mixed model (LMM) to a time-series of census-level community-weighted mean for each trait. The models consider plot intercept and slope as a random effects. In bold slopes that significantly differ from zero, considering $\alpha = 0.05$.

Functional type	Intercept	Slope (ha^{-1} y^{-1})	Relative change % ha^{-1} y^{-1}
small pioneers	0.04	$-2x10^{-5}$	-0.04
small non-pioneers	0.03	**$-9x10^{-5}$**	**-0.29**
tall pioneers	0.10	$-7\ x10^{-6}$	-0.01
tall non-pioneers	0.10	$-1\ x10^{-4}$	-0.10
other taxa	0.73	$2\ x10^{-4}$	0.03

Appendix S11 – Trends for domesticated taxa

It has been hypothesized that large parts of Amazonia are influenced by 'legacy' effects of indigenous forest management, with marked impacts still visible in contemporary forest composition including the relative prevalence of domesticated species (Levis *et al.*, 2017). If so, then in the absence of such management we might expect some ecological relaxation, with community change toward reduced dominance of those species which were favoured by indigenous forest management. Thus, if most plots are still recovering from previous human-use, we would expected domesticated taxa to be declining and for any declines to be greater than those of non-domesticated taxa. To test for this prediction, we investigated whether there has been a change in the proportion of domesticated species *sensu* Levis *et al.* (2017) within the plots.

We use the same approach used in the main text when analysing changes in functional composition (see *Trends in functional composition* section for details) but here testing for temporal trends in the proportion of domesticated species within the inventory plots. Firstly, we calculated the bootstrapped mean and 95% CI of linear slopes of the percentage of domesticated species as a function of time across all plots. Secondly, we used linear mixed effect model (LMM) where the percentage of domesticated species were a function of time using function *lmer* from the R package *lme4* (Bates *et al.*, 2014). Finally, we analysed each domesticated species individually based on their trends in abundance (details in the section *Trends in floristic composition section* in the main text).

Overall, the proportion of individuals of domesticated species did not change over time. This was consistent regardless the analytical method: the LMM showed 0.7% intercept, slope = 5×10^{-4} % y^{-1}; P-value = 0.35; bootstrap indicated a non-significant trend of 4×10^{-4} % y^{-1}(CI: – 5×10^{-4}; 1×10^{-3} % y^{-1}). When analysed individually we also failed to detect a decrease in domesticated taxa. Levis et al reported 85 woody species to be domesticated by pre-Columbian peoples, of which 63 are in our dataset. The results from the bootstrap analyses indicate 27 species increasing in abundance (only 5 significantly) (Table S11.1). The same analyses show 36 species decreasing in abundance, only 4 significantly (Table S11.2). These results were consistent with the outputs form the LMM analyses.

Table S11.1 – Domesticated species *sensu* Levis *et al.* (2017) that increased significantly in abundance across the Amazon Basin. Columns represent: species identity, position in the ranking of dominance across Amazonia, number of plots in which the species was found within Core Amazonia, bootstrapped mean and 95% CI (in brackets) of trends in relative abundance, intercept, slope, and percentage change per year (in brackets) from linear mixed model (LMM) fitted to a time-series of census level relative abundance of each species. The LMM considers plot slope and intercept as random effects. Values in bold and followed by + represent slopes that significantly differ from zero, considering respectively α = 0.05 and α = 0.1. Sample size was too small to precisely generate LMM for *Platonia insignis*.

Species	Hyperdom.	n. plots	Rel. abundance (% y^{-1})	Intercept	Slope
Euterpe precatoria	1	54	$2x10^{-4}(7x10^{-5}\|4x10^{-4})$	0.01	**$2x10^{-4}$ (2.2)**
Hevea brasiliensis	14	7	$2x10^{-4}(7x10^{-5}\|3x10^{-4})$	0.02	**$2x10^{-4}$ (1.2)**
Theobroma speciosum	35	35	$5x10^{-5}(2x10^{-5}\|8x10^{-5})$	$2x10^{-3}$	**$5x10^{-5}$ (2.6)**
Caryocar villosum	1204	6	$1x10^{-5}(2x10^{-6}\|3x10^{-5})$	$1x10^{-3}$	$1x10^{-5}$ (0.9)
Platonia insignis	1418	3	$4x10^{-5}(6x10^{-6}\|6x10^{-5})$		

Table S11.2 – Domesticated species *sensu* Levis *et al.* (2017) which decreased significantly in abundance across the Amazon Basin, as for A7.1. Sample size was too small to precisely generate LMM for *Eugenia uniflora* and *Genipa americana*.

Species	Hyperdom.	n. plots	Rel. abundance (% y^{-1})	Intercept	Slope
Oenocarpus bataua	7	45	$-1x10^{-4}(-2x10^{-4}\|-2x10^{-5})$	0.02	**$-2x10^{-4}$ (-1)**
Grias neuberthii	531	6	$-7x10^{-5}(-1x10^{-4}\|-3x10^{-6})$	0.01	$-7x10^{-5}$(-0.6)+
Genipa americana	1010	4	$-6x10^{-5}(-1x10^{-4}\|-2x10^{-5})$		
Eugenia uniflora	2699	5	$-2x10^{-5}(-3x10^{-5}\|-4x10^{-6})$		

References

Allen RG, Smith M, Pereira LS, Perrier A (1994) An update for the calculation of reference evapotranspiration. ICID Bulletin*,* **43**, 35-92.

Aragao L, Malhi Y, Roman-Cuesta RM, Saatchi S, Anderson LO, Shimabukuro YE (2007) Spatial patterns and fire response of recent Amazonian droughts. Geophysical Research Letters*,* **34**.

Bates, Maechler, Bolker, Walker (2014) lme4: Linear mixed-effects models using Eigen and S4. R package version 1.1-7 pp Page.

Brienen RJW, Phillips OL, Feldpausch TR *et al.* (2015) Long-term decline of the Amazon carbon sink. Nature*,* **519**, 344-348.

Chave J, Coomes D, Jansen S, Lewis SL, Swenson NG, Zanne AE (2009) Towards a worldwide wood economics spectrum. Ecology Letters*,* **12**, 351-366.

Chave J, Réjou-Méchain M, Búrquez A *et al.* (2014) Improved allometric models to estimate the aboveground biomass of tropical trees. Global Change Biology*,* **20**, 3177-3190.

Esquivel-Muelbert A, Baker TR, Dexter KG *et al.* (2017) Seasonal drought limits tree species across the Neotropics. Ecography*,* **40**, 618-629.

Fauset S, Johnson MO, Gloor M *et al.* (2015) Hyperdominance in Amazonian forest carbon cycling. Nature Communications*,* **6**.

Fyllas NM, Quesada CA, Lloyd J (2012) Deriving Plant Functional Types for Amazonian forests for use in vegetation dynamics models. Perspectives in Plant Ecology, Evolution and Systematics*,* **14**, 97-110.

Harris I, Jones PD, Osborn TJ, Lister DH (2014) Updated high-resolution grids of monthly climatic observations – the CRU TS3.10 Dataset. International Journal of Climatology*,* **34**, 623-642.

Huffman GJ, Bolvin DT, Nelkin EJ *et al.* (2007) The TRMM Multisatellite Precipitation Analysis (TMPA): Quasi-Global, Multiyear, Combined-Sensor Precipitation Estimates at Fine Scales. Journal of Hydrometeorology*,* **8**, 38-55.

Levis C, Costa FRC, Bongers F *et al.* (2017) Persistent effects of pre-Columbian plant domestication on Amazonian forest composition. Science*,* **355**, 925-931.

Lewis SL, Lopez-Gonzalez G, Sonke B *et al.* (2009) Increasing carbon storage in intact African tropical forests. Nature*,* **457**, 1003-U1003.

Zanne AE, Westoby M, Falster DS, Ackerly DD, Loarie SR, Arnold SEJ, Coomes DA (2010) Angiosperm wood structure: Global patterns in vessel anatomy and their relation to wood density and potential conductivity. American Journal of Botany*,* **97**, 207-215.
